# Combined transcriptome and proteome analysis of Bcfrp1 involved in regulating the biosynthesis of abscisic acid and growth in *Botrytis cinerea* TB-31

**DOI:** 10.3389/fmicb.2022.1085000

**Published:** 2023-01-26

**Authors:** Dongbo Chen, Dan Shu, Zhao Wei, Di Luo, Jie Yang, Zhemin Li, Hong Tan

**Affiliations:** ^1^CAS Key Laboratory of Environmental and Applied Microbiology, Environmental Microbiology Key Laboratory of Sichuan Province, Chengdu Institute of Biology, Chinese Academy of Sciences, Chengdu, China; ^2^Chengdu Institute of Biology, China Academy of Sciences (CAS), University of the Chinese Academy of Sciences, Chengdu, China

**Keywords:** *bcfrp1*, *Botrytis cinerea*, abscisic acid, carbon metabolism, transcriptome, proteome

## Abstract

**Introduction:**

Abscisic acid (ABA) is an important sesquiterpene compound that regulates the stress resistance of plants. *Botrytis cinerea* can synthesize ABA via the mevalonic acid pathway. To identify the functional genes that are involved in the biosynthesis of ABA, we performed insertion mutagenesis into *B. cinerea* TB-31.

**Methods:**

We obtained the ABA-reduced mutant E154 by insertion mutagenesis, and we identified the insertion site was located upstream of the gene *bcfrp1* by Thermal asymmetric interlaced PCR. We performed a detailed phenotypic characterization of the *bcfrp1* knockout and complementation mutants in TB-31. Furthermore, transcriptome and proteome analyses were conducted to explore how *bcfrp1* affects the level of the ABA biosynthesis.

**Results:**

The *bcfrp1* gene encodes an F-box protein. The phenotypic results confirmed the positive contribution of *bcfrp1* to the biosynthesis of ABA and growth. Between TB-31 and ΔBcfrp1, we obtained 4,128 and 1,073 differentially expressed genes and proteins, respectively. The impaired ABA biosynthesis in the ΔBcfrp1 mutants was primarily affected by the different levels of expression of the ABA biosynthetic gene cluster and the genes involved in the mevalonic acid pathway. In addition, we further characterized the differentially expressed genes and proteins that participated in the growth, secondary metabolism, and signal transduction in *B. cinerea* based on the transcriptome and proteome data.

**Discussion:**

This research based on the transcriptome and proteome analyses to display the changes after the deletion of *bcfrp1* in *B. cinerea* TB-31, will help us to explore the molecular mechanism of ABA biosynthesis in *B. cinerea*.

## 1. Introduction

Abscisic acid (ABA) is a sesquiterpene “stress hormone,” which has been shown to be synthesized not only in plants, but also in cyanobacteria, fungi, sponge, human cells amongst others (Gill and Patranabis, 2021). The most common functions of ABA are involved in controlling seed maturation and germination, increasing the tolerance of plants for various kinds of stresses caused by abiotic or biotic factors (Alazem and Lin, 2017). In addition, ABA has also demonstrated its important functions in other organisms (Sakthivel et al., 2016). It plays important roles in regulating the activation of innate immune cells and glucose homeostasis in mammals (Gietler et al., 2020). Interestingly ABA also seems to exist as a communication signal between different species (Lievens et al., 2017). There are reports of some mutualistic host-microbe and host-pathogen interactions dependence on ABA (Lievens et al., 2017; Hewage et al., 2020). For these reasons, it is very important to understand the biosynthesis and regulation mechanism of ABA in non-plant organisms. It will be very interesting to elucidate the biological significant of ABA as a ‘universal molecule’.

Phytopathogenic fungi, such as *Botrytis cinerea* has been shown to produce ABA. Marumo et al., first reported that the gray mold fungus *B. cinerea* could synthesize ABA in 1982 (Marumo et al., 1982). After that, other *B. cinerea* strains have been confirmed and considered to be ABA-producing strains (De Simone et al., 2020). Twenty years ago, our research group isolated a wild type strain *Botrytis cinerea* TBC-6 from the stems and leaves of wheat in southwest China. Mutant strains TB-31(with ABA productivity of 0.55 g/L) and TB-3-H8 (with an ABA productivity of 1.4 g/L) were generated after multiple rounds of mutagenesis and screening of TBC-6 (Gong et al., 2014; Wang et al., 2018). Further strain improvement generated the TBC-A strain with an ABA productivity of 2.0 g/L, which has been utilized to reduce the cost of ABA in industrial applications (Gong et al., 2014; Ding et al., 2015). However, little is known about in the molecular mechanisms underlying fungal ABA biosynthesis before these strains can be used in an industrial setting for biotechnological ABA production (Ding et al., 2016).

^18^O-, ^2^H-, and ^13^C-labeling experiments were performed to study the ABA biosynthetic pathway of *B. cinerea*, and a pathway different from plants has been postulated: isopentenyl pyrophosphate (IPP), which is synthesized from the mevalonic acid (MVA) pathway, is converted to C_15_ compound farnesyldiphosphate (FPP). Additionally, after a series of reactions of cyclization, isomerization, desaturation and hydroxylation from FPP, ABA is synthesized (Nambara and Marion-Poll, 2005; Siewers et al., 2006). But there was no genetic information to support this hypothesis, and it was not until 2004 that genes involved in ABA synthesis were discovered. The gene knockout experiments revealed the *bcaba1-4* gene cluster, in which *bcaba1*, *2*, *4* genes were presumed to be responsible for the hydroxylation of carbon atoms C-4′, C-1′ and the oxidation of C-4′ of ABA in *B. cinerea*, respectively (Siewers et al., 2004, 2006).

In order to further understand the synthesis and regulatory mechanisms of ABA, we had carried out systematic research in the previous work. First, Ding et al. (2016) performed a comparative transcriptome analysis to identify the differentially expressed genes which may potentially contribute to the very different phenotypic outcomes of ABA production of the industrial strain TBC-A and the wild-type strain TBC-6. The results of comparative transcriptome analysis helps us understand the crucial genes and metabolic pathways related to ABA biosynthesis, including genes involved in the MVA pathway and the biosynthesis of FPP, which further confirmed that the ABA synthesis pathway of fungi is different from that of plants (Ding et al., 2016). Second, the genes that are required for ABA production and regulation were identified based on the transcriptome data, such as *bcaba3*, *bcabaR1*, and *bclaeA*. In our previous study, the targeted disruption of Bcaba3 showed the loss of ABA synthesis in TB-31, and enabled the detection of 2Z,4E-α-ionylideneethane (data not published). Shu et al. (2018) found that Bcaba3 can catalyze the substrate FPP into 2Z,4E-α-ionylideneethane through the enzyme catalysis experiments, which is consistent with the report of Takino et al. (2018). Wang et al. (2018) reported a Cys_2_His_2_ Zinc finger transcription factor BcabaR1 which positively regulates the levels of transcription of *bcaba1-4*, by interacting with conserved sequence regions of the ABA gene clusters promoters. Wei et al. (2022) found the global regulator BcLaeA, as ‘a putative methyltransferase’, also participates in regulating the biosynthesis of ABA in TB-31 (Wei et al., 2022). Third, in order to find some new unknown genes that may be involved in ABA synthesis, we also performed insertional mutagenesis in *B. cinerea* TB-31 by *Agrobacterium tumefaciens*-mediated transformation (ATMT; Chen et al., 2011), which is a useful approach that enables the identification of functional genes.

Plasmid insertion induction is a common method for identifying new genes or discovering new functions of the known gene (Miesel et al., 2003). Duyvesteijn et al. (2005) obtained a pathogenic deletion mutant from *Fusarium oxysporum* f. sp. *lycopersici* by random insertion, and identified that the insertion site occurred in a gene encoding an F-box protein and named Frp1 (Duyvesteijn et al., 2005). It was reported that F-box protein (FBP) is part of SCFs (Skp1-cullin-F-box protein ligase) complexes and linked to the Skp1 protein through the F-box (Gabriel et al., 2021). Of course, F-box proteins function as non-SCF complexes, too (Sadat et al., 2021; Wu et al., 2022). Each eukaryote contains a considerable amount of F-box proteins, and each protein may correspond to a substrate. Lots of fungal F-box protein were revealed, which have been shown to be involved in stress response, catabolite repression, and to be a novel virulence factor that shapes immunogenicity and such (De Assis et al., 2018; Masso-Silva et al., 2018; Sharma et al., 2021). However, there are few reports about the effects of F-box protein on secondary metabolic synthesis in fungi.

In this research, we report an ABA deficient mutant *B. cinerea* E154 from an ATMT-mediated random mutation, and the F-box protein Bcfrp1 was identified which was responsible for the phenotype of E154. It has not been reported that ubiquitin-related proteins are involved in the regulation of ABA synthesis in *B. cinerea*. The role of Bcfrp1 in ABA biosynthesis and the growth of *B. cinerea* was investigated. A stringent comparative transcriptome and proteome analysis was performed to identify differentially expressed genes participating in the metabolic pathways related to ABA biosynthesis and other pathways in *B. cinerea*. This study could help us to predict and screen genes related to the effect of *bcfrp1* on ABA biosynthesis in *B. cinerea* based on the transcriptome and proteome.

## 2. Materials and methods

### 2.1. Strains, plasmids, and culture conditions

*Escherichia coli* DH5α and *Agrobacterium tumefaciens* EHA105 were used to construct vectors and transform plasmid DNA. *E. coli* and *A. tumefaciens* strains were grown on Luria-Bertani (LB) media. The plasmid pBHt2 enables the selection of hygromycin-resistant (*hph*) transformants. The ABA-producing strain *B. cinerea* TB-31 was transformed with pBHt2 to create insertional mutants by ATMT (Rolland et al., 2003)*. B. cinerea* TB-31 was used as a control for this study to generate gene knockout and complementary mutants of *bcfrp1*. *B. cinerea* strains grow on potato glucose agar (PDA) media at 26°C for 7 days, followed by the collection of spores and resuspended to 1 × 10^6^ conidia·mL^−1^. The PDA plates with 50 μg·mL^−1^ hygromycin B or 100 μg·mL^−1^ glufosinate-ammonium, were purchased from Solar Biotech, to screen the *B. cinerea* transformants.

### 2.2. Construction of the deletion and complementation mutants of *bcfrp1*

We generated knockout and complementary mutants of *bcfrp1* to explore whether this gene affected the synthesis of ABA in *B. cinerea* TB-31. Lists of all the primers used to prepare these constructs are shown in Supplementary Table S1. The *bcfrp1* gene knockout vector was constructed using the double-joint PCR method (Yu et al., 2004). The genomic DNA of *B. cinerea* TB-31 was extracted by the E.Z.N.A. Fungal DNA Mini kit (Omega, D3390-01, United States). With the DNA of TB-31 as the template, we amplified the 5′ fragment (552 bp) and 3′ fragment (613 bp) of the open reading frame (ORF) of *bcfrp1* by the primer pairs *bcfrp1*-5′F/-R and *bcfrp1*-3′F/-R, respectively. The *hph* fragment was amplified using primer pairs *hph*-F/-R. With the primer pair *bcfrp1*-5′F/3′-R to amplify the gene knockout cassette by overlapping PCR (Supplementary Figure S2A). The gene knockout cassettes were transformed into fungal protoplasts as described by Choquer et al. (2008). Transformants were selected on PDA containing hygromycin (50 μg·mL^−1^), and three generations were purified on PDA with hygromycin. Transformants were verified by PCR using the primers *bcfrp1*-out-5′F/*hph*-in-R, *hph*-in-F/*bcfrp1*-out-R, *hph*-F/-R, *bcfrp1*-detcet-F/-R which were shown in Supplementary Table S1, and driven by real-time quantitative reverse transcription PCR (qRT-PCR) using the primers qRT-*bcfrp1*-F/-R.

To further confirm that the differences in production of ABA between ΔBcfrp1 and TB-31 were caused by the loss of *bcfrp1*, we constructed the complementary vector pCBg1-*bcfrp1* and then transformed it into a ΔBcfrp1 mutant (Supplementary Figure S2A). We amplified *bcfrp1* cDNA from *B. cinerea* TB-31 using the primers *bcfrp1*-cds-F/−R, and cloned into the *Sca*I and *Xba*I digested sites in pCBg1 to generate pCBg1-*bcfrp1*. The vector, pCBg1-*bcfrp1*, was utilized for ATMT of the conidia of ΔBcfrp1. Transformants were selected on PDA that contained hygromycin (50 μg·mL^−1^) and glyphosate (100 μg·mL^−1^), and three generations were purified on PDA with hygromycin and glyphosate. The transformants were verified by PCR using the primers *bcfrp1*-detcet-F/-R, *hph*-F/-R, Bar-in-F/-R, and qRT-PCR using the primers qRT-*bcfrp1*-F/-R.

### 2.3. Phenotypic analysis of the deletion and complementation mutants of *bcfrp1*

To detect the extracellular ABA in TB-31 and the ΔBcfrp1 and ΔBcfrp1-C mutants, these strains were inoculated on PDA media for 6–12 days at 26°C. The detection of ABA yield was performed by high-performance liquid chromatography (HPLC) as described by Ding et al. (2015). The biomass was quantified by collecting the mycelia of the three strains from 50 ml of PDB after 72, 96 and 120 h. The mycelia were filtered and weighed after drying at 100°C for 60 min (Ding et al., 2016). The colony diameters of the TB-31 and mutant strains were analyzed on agar plates. Briefly, each strain was separately inoculated on Czapek–Dox agar (CDA) media with different sugar carbon sources: 2% D-glucose, 2% sucrose, 2% lactose, or 2% maltose, respectively, for 6 days at 26°C. Each strain was analyzed in triplicate.

### 2.4. Transcriptome sequencing and analysis

After culturing on PDA in the dark for 6 days at 26°C, the mycelia of the *B. cinerea* TB-31 and ΔBcfrp1 were harvested and stored at −80°C. The total RNA was extracted using TRIzol according to the manufacturer’s instructions (Invitrogen, Carlsbad, CA, United States). After the quality and concentration of RNA were tested and qualified, the RNA library was sequenced by Gene Denovo Biotechnology Co., Ltd. (Guangzhou, China) with the Illumina NovaSeq 6,000 (San Diego, CA, United States; Modi et al., 2021). The raw data that were obtained were filtered and mapped to the genome of model microorganism *B. cinerea* B05.10 (NCBI, ASM14353v4) using HISAT2.2.4 (Kim et al., 2015). The genes obtained were quantified using RNA-seq by expectation maximization (RSEM; Li and Dewey, 2011), and their levels of expression were normalized using Fragments Per Kilobase of transcript per Million mapped reads (FPKM) as described by Li and Dewey (2011). The differential expression analysis was analyzed between TB-31 and ΔBcfrp1 by DESeq2 (Love et al., 2014). The differentially expressed genes (DEGs) were filtered based on fold change ≥2 and false discovery rate (FDR) ≤0.05 (FPKM > 1). The gene functions were annotated against the cluster of orthologs/eukaryotic clusters of orthologs (COG/KOG), Gene Ontology (GO) and Kyoto Encyclopedia of Genes and Genomes (KEGG) database as described by Fang et al. (2021).

### 2.5. Proteome analysis

Consistent with the samples analyzed by transcriptome sequencing, the samples for proteome analysis were also harvested after 6 days of culture on PDA. The total protein of TB-31 and ΔBcfrp1 was extracted and measured with BCA (Bicinchoninic acid) method as described by Jia et al. (2022). The proteins were digested into peptide fractions and performed with the method of data-independent acquisition (DIA) as described by Jia et al. (2022). DIA raw data were performed by Spectronaut X (Biognosys AG, Schlieren, Switzerland) as described by Long (Long et al., 2020). All the selected precursors passing the filters were employed to quantify. The differentially expressed proteins (DEPs) were filtered based on fold change ≥1.5 and *p* < 0.05 after subjecting them to Student’s *t*-test. The protein function were annotated against COG/KOG, GO and KEGG database with *p* ≤ 0.05 as described by Wei et al. (2022).

### 2.6. Correlation analysis of genes and proteins

We have uploaded the Transcriptome and Proteome data in the ScienceDB (https://www.scidb.cn/detail?dataSetId=bf230977945c4b4785ec355293b23d79). First, the differentially expressed genes and proteins were identified between TB-31 and ΔBcfrp1. A significant DEG was screened with fold change ≥2 and FDR <0.05, and a significant DEP was screened with fold change >1.5 and *p* < 0.05, respectively. Second, the correlation analysis between genes and proteins were analyzed as described by Dou et al. (2022). The Venn diagram was used to count the genes/proteins and the differential genes/differential proteins, respectively. The correlation analysis between genes and proteins were carried out by R language (version 3.5.1). In addition, the nine-quadrant map shows the functional enrichment with the GO and KEGG pathway analysis of mRNAs and proteins.

### 2.7. qRT-PCR analysis

All the mycelial RNA was extracted using TRIzol according to the manufacturer’s instructions (Invitrogen, Carlsbad, CA, United States). The quality and concentration of RNA was detected as described by Wang et al. (2018). cDNA was synthesized by the TaKaRa reverse transcriptase kit (TaKaRa, Dalian, China). The qRT-PCR was conducted using the AceQ qPCR SYBR Green master mix kit (Nanjing Vazyme Biotech Co., Ltd., Nanjing, China). The qRT-PCR primers in this study are shown in Supplementary Table S1. The reactions were conducted in a CFX96 real-time PCR detection system (Bio-Rad, Hercules, CA, United States). Each transcript was analyzed using three PCR repetitions in parallel. The tubulin gene (Bcin02g00900) served as an internal control (Dulermo et al., 2010). The fold change in the mRNA was analyzed by 2^−ΔΔCt^ method.

## 3. Results

### 3.1. Identification ABA-deficient mutant named E154

*Botrytis cinerea* TB-31 was transformed with pBHt2, which is a commonly used binary vector for ATMT in filamentous fungi, to create ABA-deficient mutants by insertional mutagenesis. This plasmid enables the selection of hygromycin-resistant (*hph*) transformants. The ATMT resulted in 217 hygromycin-resistant transformants that were grown for 8 days on PDA media and tested for their production of ABA using HPLC. Among the positive transformants, one mutant designated E154 exhibited a reduction of approximately 40% in the yield of ABA compared with the parental strain TB-31 when cultured on PDA agar for 6 days (Supplementary Figure S1A). However, the E154 mutant had a similar colony morphology when compared with TB-31 (Supplementary Figure S1B).

Thermal asymmetric interlaced PCR (TAIL-PCR) was carried out to isolate the portion of the genome that is close to either the right or left boundary of the T-DNA integration event as described from rice blast (*Magnaporthe oryzae*; Chen et al., 2011). In this study, we found that T-DNA was integrated in TB-31 at 1806 bp upstream of the predicted start codon of BC_5_05922 (Supplementary Figure S1C; Supplementary Table S2). A reverse transcription (RT)-PCR assay showed that the level of transcription of BC_5_05922 decreased significantly in the E154 mutant (Supplementary Figure S1D). The full-length DNA and cDNA were also isolated for BC_5_05922 from TB-31, and the complementary assay showed that the reduction of ABA could be complemented in *trans* by integrating a functional copy of the BC_5_05922 cDNA into the E154 strain using the vector pCBg1 (Supplementary Figure S1A). In addition, it showed that the translated protein of BC_5_05922 had 100% amino acid identity with Bcfrp1 (BCIN_11g00230) of *B. cinerea* B05.10 using the BLASTP algorithm from NCBI. These findings suggested that T-DNA integration upstream of the *bcfrp1* gene may be linked to the E154 phenotype.

### 3.2. *bcfrp1* is required for the biosynthesis of ABA in *Botrytis cinerea* TB-31

**Figure 1:**
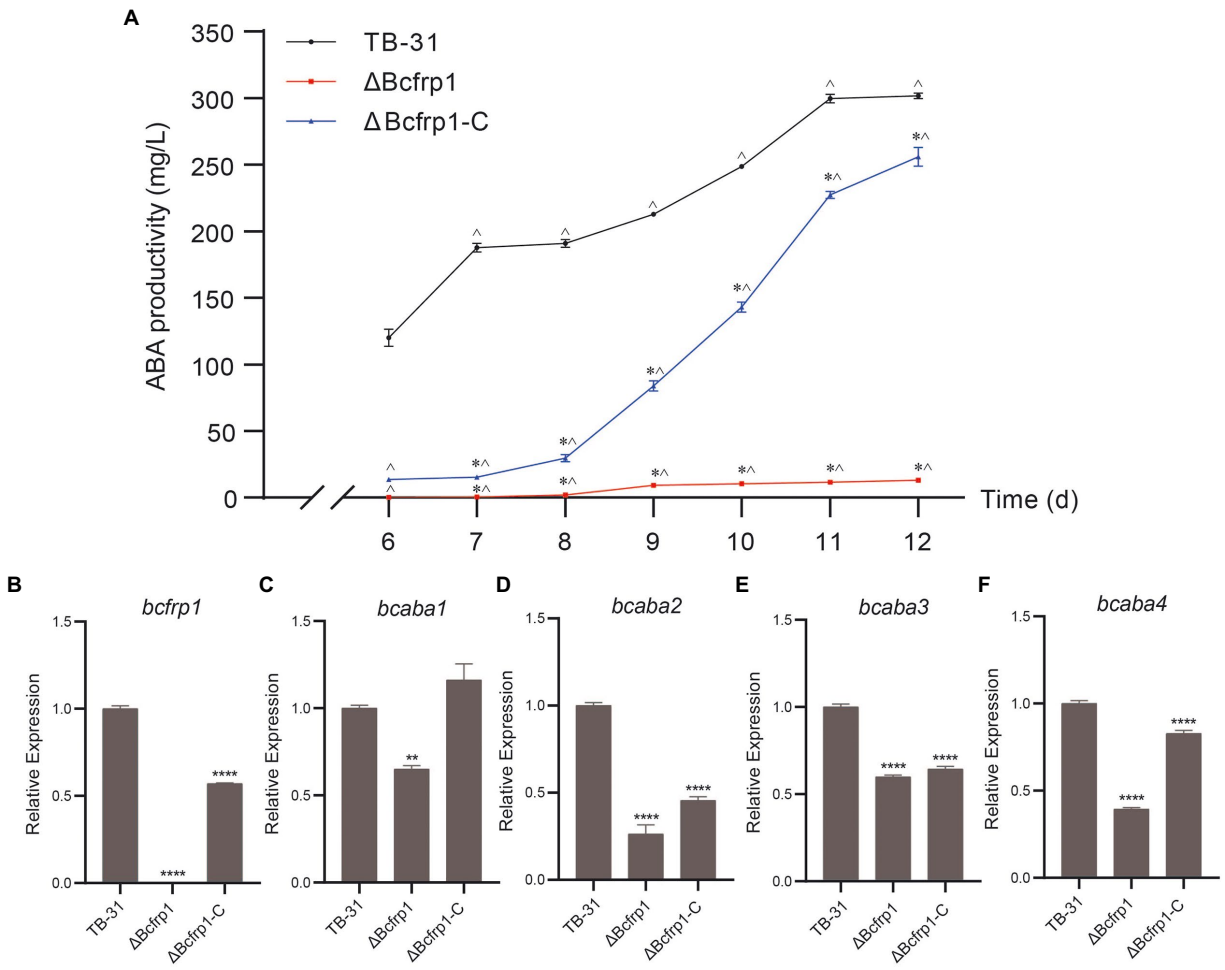
*Bcfrp1* is required for ABA yield in *Botrytis cinerea* TB-31. **(A)** ABA production of *B. cinerea* TB-31, ΔBcfrp1 and ΔBcfrp1-C mutant strains which were cultured on PDA plates at 26°C. Samples to test for ABA production were collected at seven time points (6, 7, 8, 9, 10, 11 and 12 days). The error bars indicate the standard errors of the means ± SEM; *n* = 3 replicate cultures. *, *p* < 0.05 versus the 6-day group; ^, *p* < 0.05 versus the TB-31 groups at the same time. **(B–F)** qRT-PCR examining the transcription levels of *bcfrp1*, *bcaba1*-*4* in mutant ΔBcfrp1, ΔBcfrp1-C and the parental strain, TB-31. Relative transcription levels of *bcfrp1*, *bcaba1*-*4* were generated after normalization to the constitutive tubulin reference gene *tubA* for 6 days. The relative values for *bcfrp1*, *bcaba1*-*4* transcription for 6 days in TB-31 were arbitrarily assigned a value of 1. Shown are means ± SEM; *n* = 3 replicate cultures. **, *p* < 0.01 versus the transcription level of the TB-31 group; ****, *p* < 0.001 versus the transcription level of the TB-31 group.

To confirm that *bcfrp1* is required for the biosynthesis of ABA by *B. cinerea* TB-31, we knocked out the *bcfrp1* gene and replaced the endogenous *bcfrp1* ORF with the *hph* gene. Moreover, we performed qRT-PCR and diagnostic PCR assays to confirm the *bcfrp1* gene was disrupted in the ΔBcfrp1 mutant (Supplementary Figure S2B). The productivity of ΔBcfrp1 for ABA decreased significantly by approximately 97% compared with TB-31 on PDA for 12 days (Figure 1A). To further confirm that the differences in the amount of ABA produced between TB-31 and ΔBcfrp1 was due to the loss of *bcfrp1*, we constructed the complementation vector pCBg1-*bcfrp1* and then transformed it into the mutant ΔBcfrp1 to generate the complementary mutant ΔBcfrp1-C (Supplementary Figure S2C). The amount of ABA produced by ΔBcfrp1-C returned to 85% of that of the control, TB-31, when cultured on a PDA plate for 12 days (Figure 1A).

Furthermore, the qRT-PCR trial verified that *bcfrp1* was disabled by deleting in the mutant ΔBcfrp1. The levels of transcription of *bcfrp1* in ΔBcfrp1-C were restored to approximately 57% of that produced by TB-31 when grown on a PDA plate for 6 days (Figure 1B). To investigate whether *bcfrp1* was involved in the regulation of ABA gene clusters, we conducted qRT-PCR to analyze the levels of transcription of *bcaba1-4* among the *B. cinerea* strains (Figures 1C–F). The levels of transcription of the *bcaba1*-*4* in ΔBcfrp1 were decreased to 75, 26, 60 and 40% of TB-31, respectively (Figures 1C–F). Each level of gene expression increased when ABA performance was re-established in ΔBcfrp1-C, which suggests that the deletion of *bcfrp1* affects the transcription of *bcaba1-4*. Combining with the previous data of insertional mutagenesis confirmed that *bcfrp1* is required for the biosynthesis of ABA in TB-31.

### 3.3. Bcfrp1 disrupts the normal growth of *Botrytis cinerea* TB-31

**Figure 2:**
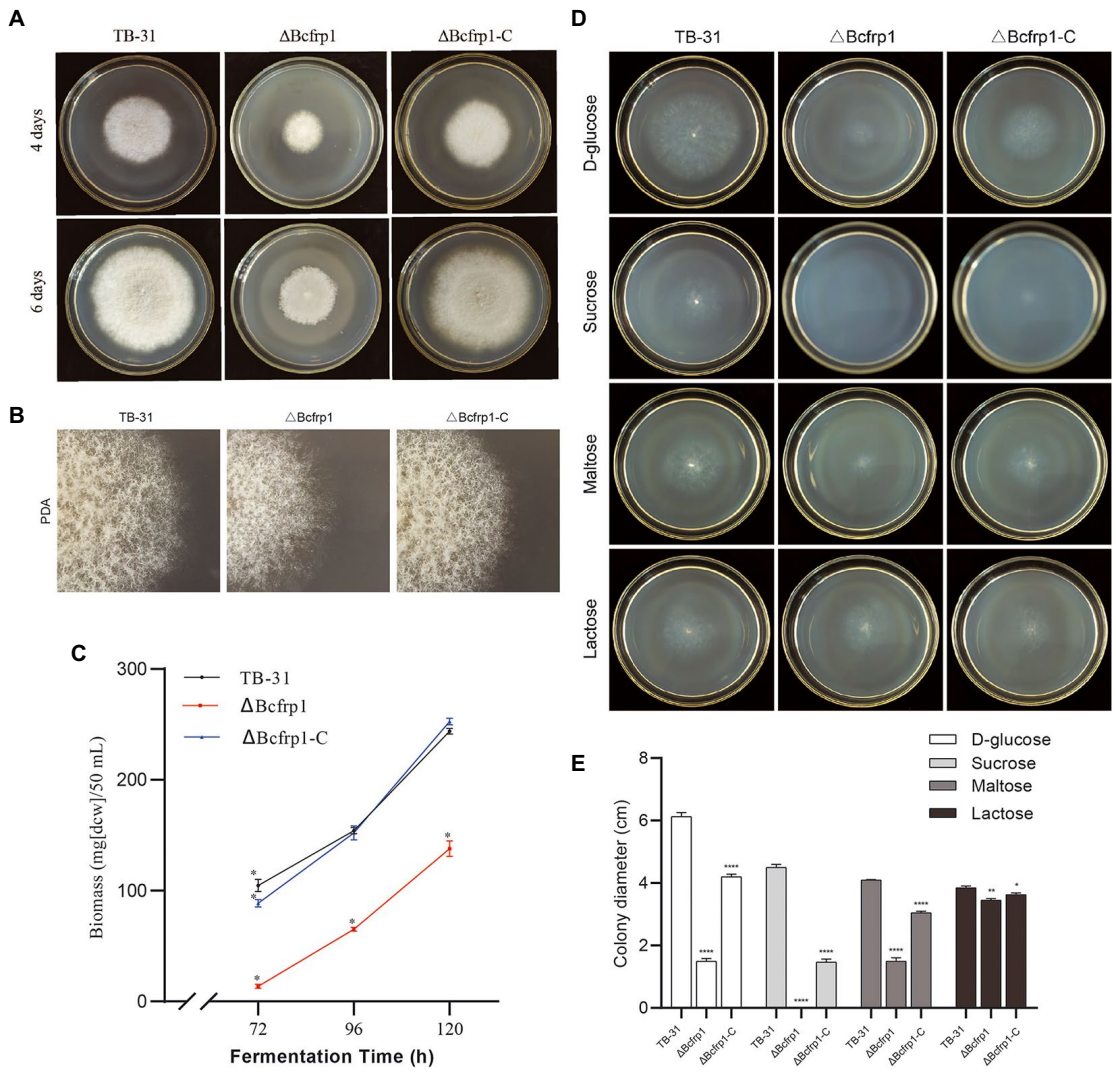
The biomass and colony morphology of *B. cinerea* TB-31, ΔBcfrp1 and ΔBcfrp1-C. **(A)** The morphology of TB-31, ΔBcfrp1 and ΔBcfrp1-C on PDA media for 4 days and 6 days. **(B)** The morphology of TB-31, ΔBcfrp1 and ΔBcfrp1-C on PDA media for 4 days and 6 days. All images were captured at 3X magnification. **(C)** Biomass of TB-31, ΔBcfrp1 and ΔBcfrp1-C on PDB media at 26°C at 180 rpm on an orbital shaker. The mycelia were collected at three time points (72, 96, and 120 h). Shown are means ± SEM; *n* = 3 replicate cultures. *, *p* < 0.05 versus the biomass of the TB-31 group. **(D)** The colony morphology of TB-31, ΔBcfrp1 and ΔBcfrp1-C on CDA media with 2% D-glucose, 2% Sucrose, 2% Maltose, and 2% Lactose plates and cultured at 26°C for 6 days. **(E)** Colony diameters of the TB-31, ΔBcfrp1 and ΔBcfrp1-C strains on CDA media with 2% D-glucose, 2% Sucrose, 2% Maltose, and 2% Lactose cultured at 26°C for 6 days. Shown is means ± SEM; *n* = 3 replicate cultures. *, *p* < 0.05 versus colony sizes of the TB-31 group; **, *p* < 0.01 versus the transcription level of the TB-31 group; ****, *p* < 0.001 versus the transcription level of the TB-31 group.

We compared the morphology and radial growth of *B. cinerea* TB-31, ΔBcfrp1, and ΔBcfrp1-C cultured on PDA. The growth rate of hyphae in ΔBcfrp1 mutant slowed down and no matter how long it takes to cultivate, the hyphae of ΔBcfrp1 could not be extended to the whole plate like TB-31. The hyphae of ΔBcfrp1 also became lighter and had sporadic whiteness. The mycelial layer of ΔBcfrp1 became thinner, with the decrease in the number of conidia compared with those of TB-31. Simultaneously, the introduction of the *bcfrp1* cDNA into ΔBcfrp1 restored the wild-type morphology and growth phenotypes, confirming that this mutant had been complemented (Figures 2A,B). The results showed that *bcfrp1* was positive for the hyphal growth of TB-31. Moreover, we compared the biomass of three strains grown on PDB media. The biomass of ΔBcfrp1 mutant decreased to 56.5% of TB-31 after 120 h of culture on PDB, while the biomass of ΔBcfrp1-C mutant was close to that of the parental strain TB-31 on PDB media (Figure 2C). These results suggested that *bcfrp1* was important for maintaining the normal growth of TB-31.

In addition, we compared the colony diameters of TB-31, ΔBcfrp1, and ΔBcfrp1-C on CDA media for 6 days with several carbon sources (Figure 2D). As shown in Figure 2E, the colony diameter of the ΔBcfrp1 mutant cultured on CDA with 2% D-glucose, 2% maltose, and 2% lactose as carbon sources was 1.5, 1.5 and 3.5 cm, respectively. Moreover, ΔBcfrp1 could not grow on CDA plates that contained sucrose. Moreover, the colony size of ΔBcfrp1 in different carbon sources could be recovered to varying degrees through complementation with *bcfrp1*.

### 3.4. The transcriptomics and proteomics analysis of *Botrytis cinerea* TB-31 and ΔBcfrp1

To further analyze the dynamic changes in the levels of expression of genes and proteins after the deletion of the *bcfrp1* gene in *B. cinerea* TB-31, mycelial samples of TB-31 and ΔBcfrp1 were collected after cultivation on PDA media for 6 days and subjected to transcriptome and proteome analyses, respectively.

**Figure 3:**
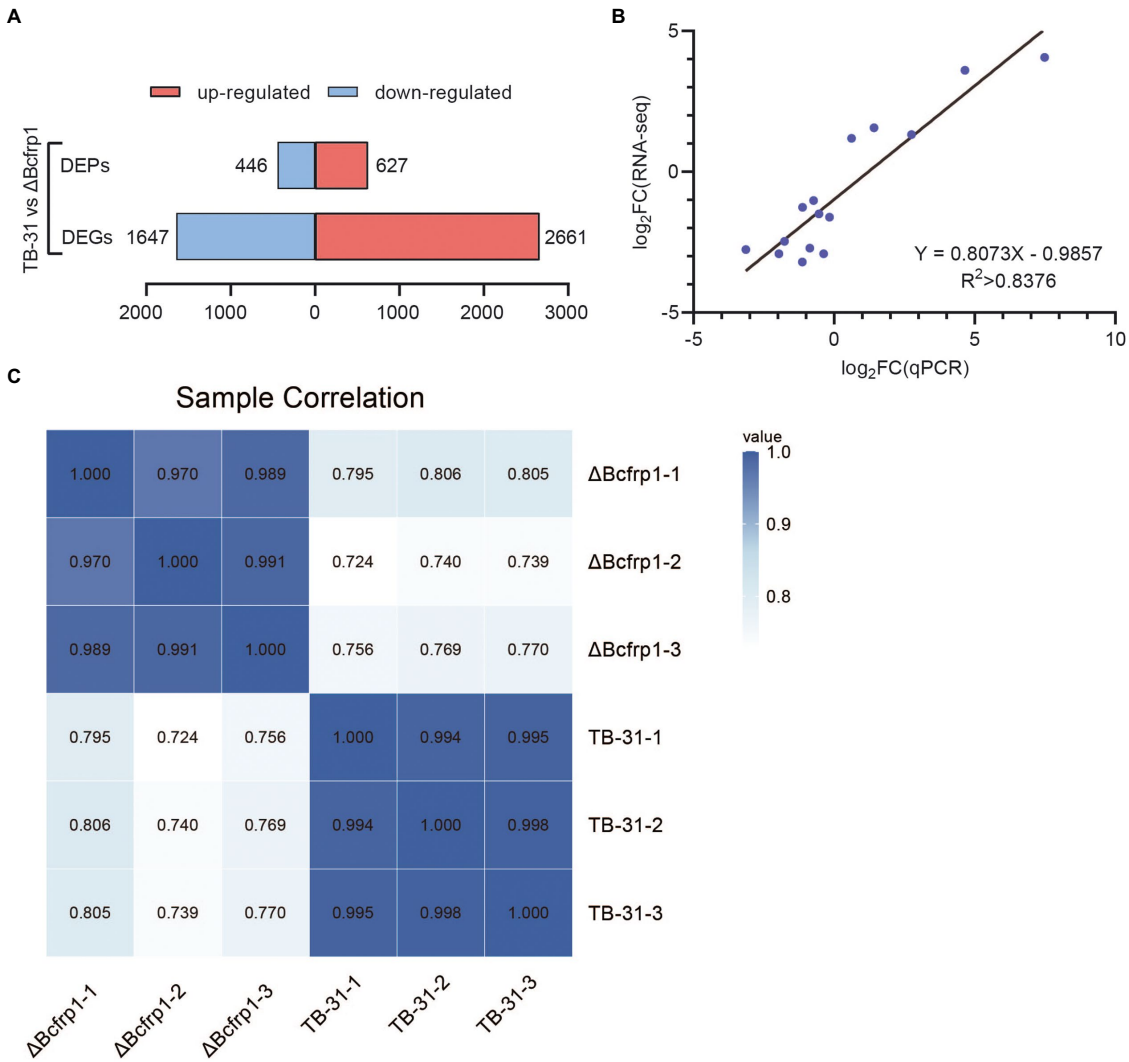
The transcriptomics and proteomics analysis of *B. cinerea* TB-31 and ΔBcfrp1. **(A)** Histogram of differential gene and protein expression among different groups. **(B)** Regression analysis of gene expression levels determined by RNA-seq and RT-qPCR (*p* < 0.05), rpearson = 0.8376. **(C)** The protein sample correlation heat map between the TB-31 and ΔBcfrp1, pearson > 0.97.

High-throughput RNA sequencing (RNA-seq) generated more than 30 million clean reads for each sample, and three biological replicates were performed for each group. As a result, 12,303 genes were detected in the library, and there are 11,707 genes in the whole genome. The other 596 genes are newly predicted genes. In total, 4,128 (2,661 upregulated and 1,647 downregulated) were identified as DEGs (log_2_ratio > 1) in TB-31 (as the control) vs. ΔBcfrp1 (Figure 3A). 15 genes were randomly selected for qRT-PCR analysis to verify RNA-seq data (Figure 3B), and the gene expression levels measured by the two methods were linearly correlated (rpearson = 0.8376).

To examine the proteins altered by *bcfrp1*, proteomic profiles were analyzed between TB-31 and ΔBcfrp1 with three biological replicates. In total, 45,527 peptides and 5,645 proteins were identified in the proteomes of TB-31 and ΔBcfrp1. A Pearson correlation analysis was highly repeatable and reliable (Pearson > 0.97) among the three biological replicates (Figure 3C). A total of 1,073 DEPs (log_2_ratio > 0.58) were observed. A total of 446 proteins were downregulated, and 627 proteins were upregulated in the mutant group compared with the TB-31 group (*p* < 0.05; Figure 3A).

### 3.5. Correlation analysis of transcriptome and proteome

**Figure 4:**
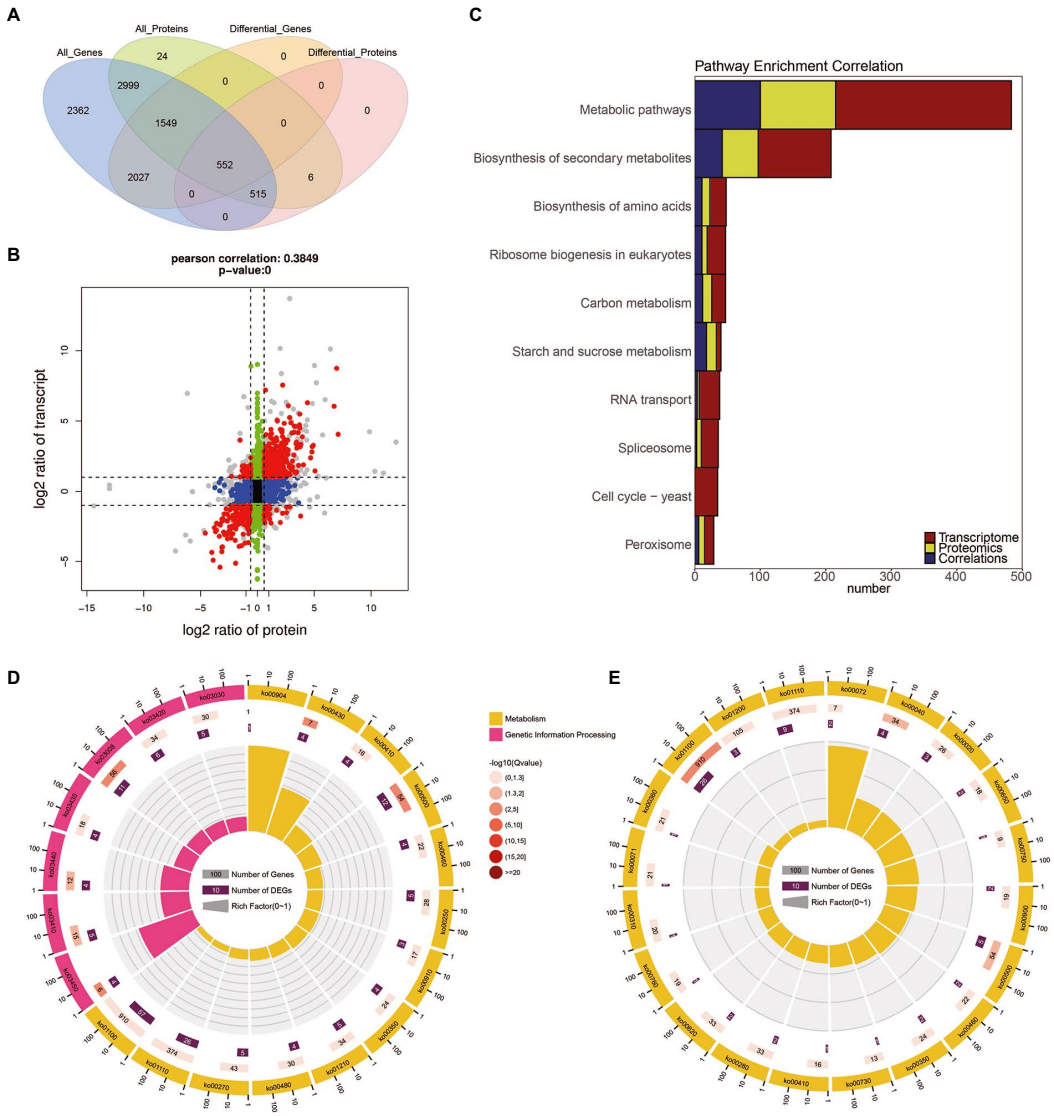
The correlation of transcriptome and proteome between the *B. cinerea* TB-31 and ΔBcfrp1. **(A)** Venn diagram of the number of all mRNAs and proteins between TB-31 and ΔBcfrp1. **(B)** The nine-quadrant graph of TB-31 and ΔBcfrp1 (considering the statistical significance, p). [The abscissa is the fold change of the protein (take ratio > 1.5), the ordinate is the fold change of the transcription (take ratio > 2), and at the top of the figure is the Pearson correlation and statistical significance, p, associated with the transcription and proteomic data. Each dot represents a gene/protein: the black dots represent non-different proteins and genes, and the red dots represent the same or opposite trend of changes in genes and proteins, and the gray dots indicate the differential expression of genes but non-differential expression of proteins]. **(C)** Histogram of the number of genes on the pathway overlapped by TB-31 and ΔBcfrp1 [The number of differentially expressed mRNA (red), protein (yellow), and associated genes (blue) is annotated on the pathway, respectively]. **(D)** The circular plot of the pathway overlapped in the quadrant 3 of the nine-quadrant map between TB-31 and ΔBcfrp1. **(E)** The circular plot of the pathway overlapped in the quadrant 7 of the nine-quadrant map between TB-31 and ΔBcfrp1.

The transcriptome and proteome data were performed with a global correlation analysis. In Figure 4A, a total of 5,615 proteins matched the transcripts, including 1,067 which were DEPs, 2,101 which were DEGs, and 552 were shared as both DEPs and DEGs. In Figure 4B, the nine-quadrant diagram shows that there are 941 NDEGs/NDEPs in quadrant 5. The 268 and 98 candidate proteins were DEGs which differentially transcribed in quadrant 1 and quadrant 9, respectively. The 81 and 86 candidate proteins were DEPs which were not differentially transcribed in quadrant 4 and quadrant 6, respectively. The 12 and 10 candidate proteins had the opposite patterns of expression from their transcripts in quadrant 1 and quadrant 9, respectively. In addition, the 103 and 32 candidate proteins showed the same expression patterns as the transcripts in the quadrants 3 and 7, respectively.

We performed pathway enrichment using the KEGG to explore the possible pathways which may be affected by *bcfrp1*. The results showed that the most DEGs/DEPs were enriched in Metabolic pathways (368 DEGs/ 215 DEPs), and the Biosynthesis of secondary metabolites (153 DEGs/ 97DEPs), and Biosynthesis of amino acids (36 DEGs/ 23 DEPs; Figure 4C). As shown in Figures 4D,E, the DEG/DEP pairs in quadrant 3 and 7 were mainly enriched in the pathways of Metabolic, Secondary metabolites, and Starch and sucrose metabolism.

To screen out the pathways that may play important roles with *bcfrp1*, we performed a pathway enrichment analysis based on the KEGG database. The results showed that the most DEGs/DEPs were involved in metabolic pathways (ko01100, 368 DEGs/ 215 DEPs), followed by the biosynthesis of secondary metabolites (ko01110, 153 DEGs/ 97DEPs), and biosynthesis of amino acids (ko01230, 36 DEGs/ 23 DEPs; Figure 4C). As shown in Figures 4D,E, the DEG/DEP pairs in quadrant 3 and 7 were mainly enriched in the metabolic pathways (ko01100), biosynthesis of secondary metabolites (ko01110), starch and sucrose metabolism (ko00500).

### 3.6. DEGs and DEPs involved in putative sugar transport and degradation

**Figure 5:**
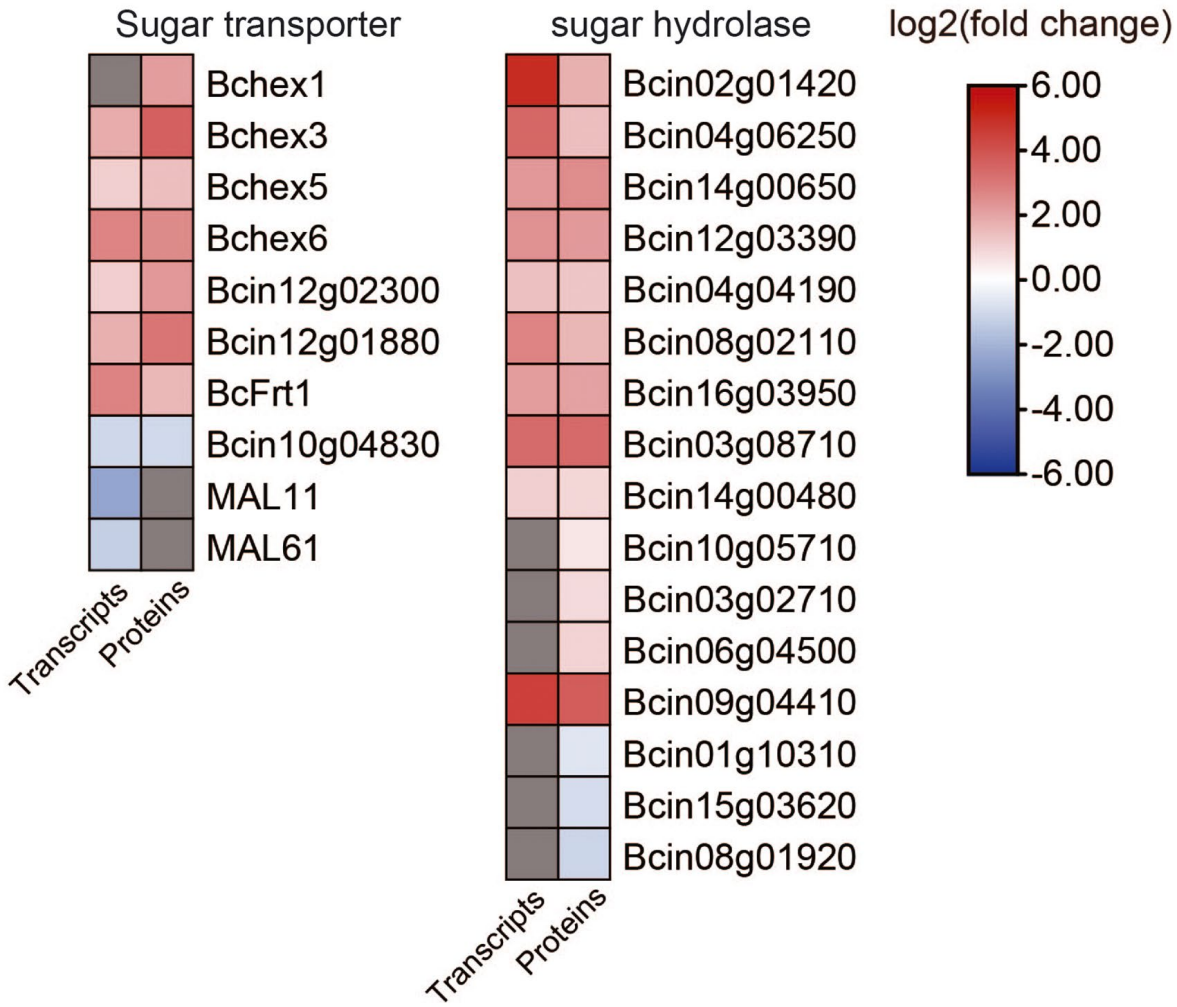
Heat map of DEGs and DEPs in sugar transport and degradation between the *B. cinerea* TB-31 and ΔBcfrp1. The heat map was generated with the software TBtools (Toolbox for Biologists) V1.0971. The gray color means no significant difference at the transcription or translation level.

We found the DEGs and DEPs involved in putative sugar transport and degradation were significantly changed for expression between *B. cinerea* TB-31 and ΔBcfrp1 (Figure 5; Supplementary Table S3).

Monosaccharide (glucose) and disaccharide (maltose, sucrose and lactose), as nutrients of fungi, can be transported to fungal cells through sugar transporters. Several genes that encode sugar transporters, including three major facilitator superfamily (MFS) sugar transporters (Bchex3, Bchex5, and Bchex6), a glucose transporter rco-3, a glucose/galactose transporter gluP, and a fructose proton symporter (BcFrt1) were upregulated at both the transcription and translation levels in ΔBcfrp1 compared with TB-31 (*p* < 0.05), while a gene that encoded an MFS sugar transporter (Bchex1) was upregulated at the translation level. In addition, a gene that encoded a protein similar to a sucrose transporter Sut1 was downregulated at both the transcription and translation levels, and two genes that encoded the maltose transporters MAL11 and MAL61 were downregulated at the transcription levels.

The transcriptome and proteome analysis also revealed that the genes that encoded two α-amylase enzymes (Bcin02g01420 and Bcin04g06250), two α-glucosidase enzymes (Bcin14g00650 and Bcin12g03390), glycoamylase (Bcgs1), and four glycoside hydrolases (Bcin08g02110, Bcin16g03950, Bcin03g08710, and Bcin14g00480) were upregulated at both the transcription and translation levels in ΔBcfrp1 compared with those of TB-31 (*p* < 0.05). Moreover, the genes that encoded a glycosyl hydrolase (Bcin10g05710), an α-galactosidase (Bcin03g02710) and a β-galactosidase (Bcin06g04500) were upregulated at the translation levels, while a galactose oxidase (Bcin09g04410) was upregulated at both the transcription and translation levels. These enzymes could be involved in the degradation of starch, sucrose, cellulose, and lactose, respectively. However, the level of translation of the genes that encoded the enzymes for glycogen degradation, such as a glycogen debranching enzyme (Bcgdb1), a glycogen phosphorylase (Bcgph1), and an ADP-sugar bisphosphatase (Bcin08g01920) were downregulated in ΔBcfrp1 compared with TB-31 (*p* < 0.05).

### 3.7. DEGs and DEPs involved in ABA biosynthesis-related pathways

**Figure 6:**
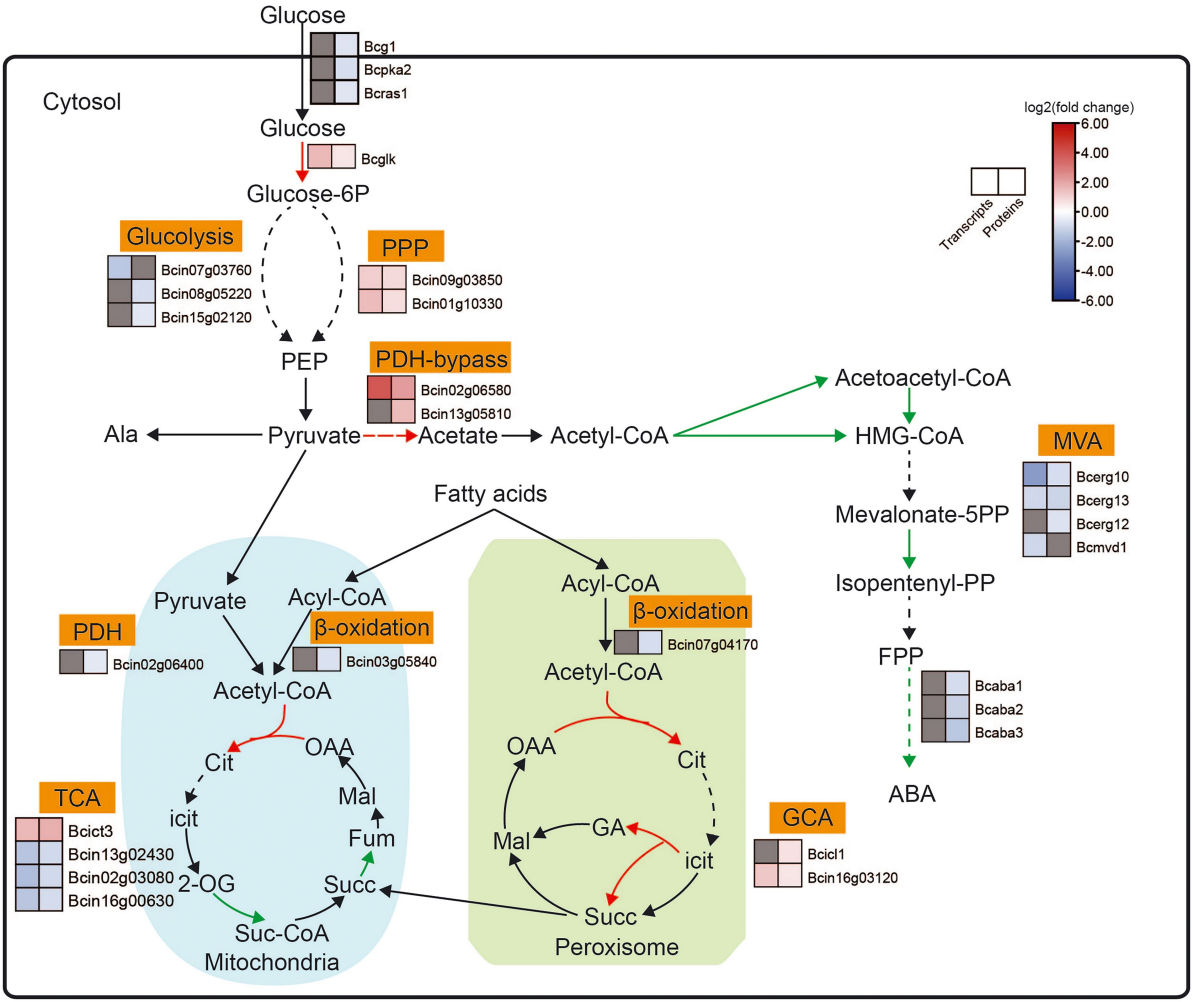
Expression pattern of key genes involved in ABA biosynthesis-related pathways between the *B. cinerea* TB-31 and ΔBcfrp1. The heat map was generated with the software TBtools (Toolbox for Biologists) V1.0971. The gray color means no significant difference at the transcription or translation level.

We analyzed the DEGs and DEPs involved in ABA biosynthesis-related pathways in *B. cinerea* TB-31 and ΔBcfrp1 (Figure 6; Supplementary Table S4).

The carbon flux from glucose to pyruvate *via* catabolic glycolysis and pentose phosphate pathway (PPP), and the genes that encoded hexokinase (Bcglk), transaldolase (Bcin09g03850) and DAHP synthetase I (Bcin01g10330) were upregulated at both the transcription and translation levels in ΔBcfrp1 compared with TB-31 (*p* < 0.05). However, the levels of translation of two genes that encoded triosephosphate isomerase (Bcin08g05220) and glyceraldehyde-3-phosphate dehydrogenase (Bcin15g02120) were downregulated, respectively. Moreover, one gene that encoded fructose-bisphosphate aldolase (Bcin07g03760); was downregulated at the transcription level in ΔBcfrp1 compared with TB-31 (*p* < 0.05). The levels of transcription of genes that encoded pyruvate decarboxylase (Bcin02g06580) and aldehyde dehydrogenase (Bcin13g05810) were upregulated in ΔBcfrp1 compared with that of TB-31 (*p* < 0.05). The results may lead to increase the production of cytosolic acetyl-CoA by the pyruvate dehydrogenase bypass. On the other hand, the genes of pyruvate dehydrogenase complex (PDH) were not significantly changed between ΔBcfrp1 and TB-31, and only one gene that encoded dihydrolipoamide dehydrogenase (Bcin02g06400) was downregulated at the translation level. Moreover, as shown in Figure 6, three genes were downregulated at the transcription and translation levels in the tricarboxylic acid (TCA) cycle. The gene that encoded ATP citrate lyase, succinate dehydrogenase, and phosphoenolpyruvate carboxykinase Bcpck1 were downregulated in ΔBcfrp1 compared with TB-31 (*p* < 0.05). In addition, a gene that encoded isocitrate lyase1 (Bcicl1) in the glyoxylate cycle (GYC) was upregulated at the translation level (*p* < 0.05).

In *B. cinerea*, acetyl-CoA can be converted to isopentenyl pyrophosphate (IPP) *via* the mevalonate (MVA) pathway, which includes seven enzymatic steps. As shown in Figure 6, three genes that encoded the acetyl-CoA synthase (Bcerg10) and 3-hydroxy-3-methylglutaryl coenzyme A synthase (Bcerg13) were downregulated at both the transcription and translation levels and mevalonate kinase (Bcerg12) was downregulated at the translation level in ΔBcfrp1 compared with TB-31 (*p* < 0.05). In addition, the level of transcription of the gene encoding MVA kinase (Bcmvd1) was also downregulated. What’s more, the expression of the ABA biosynthetic gene cluster *bcaba1*-*4* was also identified in the transcriptome and proteome of TB-31 and ΔBcfrp1. As shown in Figure 6, *bcaba1*-*3* was downregulated at the levels of translation in ΔBcfrp1 compared with TB-31 (*p* < 0.05). This suggests different levels of expression of genes involved in the MVA pathway and the ABA biosynthetic gene cluster, which may lead to ABA deficiency in ΔBcfrp1 strain.

### 3.8. DEGs and DEPs involved in secondary metabolism

**Figure 7:**
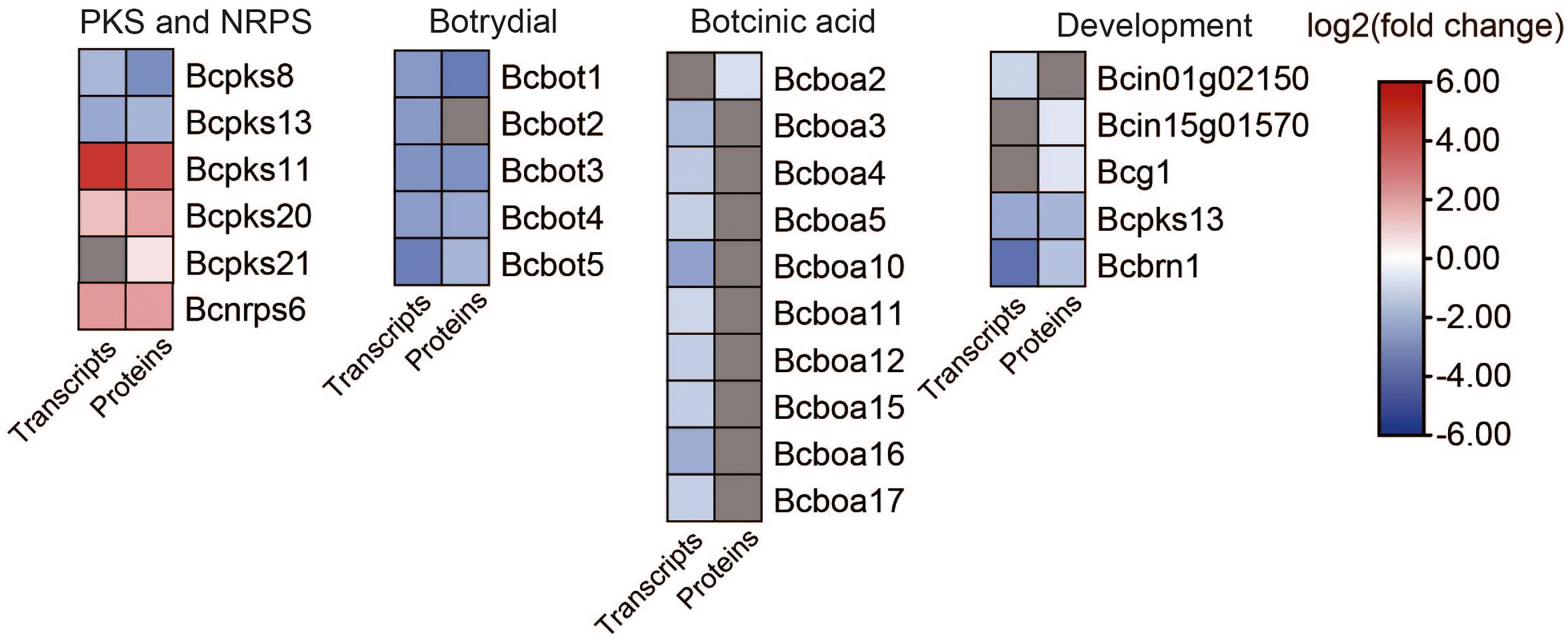
Heat map of DEGs and DEPs in secondary metabolism key enzymes encoding genes and development between the *B. cinerea* TB-31 and ΔBcfrp1. The heat map was generated with the software TBtools (Toolbox for Biologists) V1.0971. The gray color means no significant difference at the transcription or translation level.

We analyzed the DEGs and DEPs involved in secondary metabolism in *B. cinerea* TB-31 and ΔBcfrp1 (Figure 7; Supplementary Table S5).

In addition to the genes related to ABA synthesis, we also analyzed genes involved in secondary metabolism. As shown in Figure 7, five polyketide synthase (PKS) genes and one gene for a non-ribosomal peptide synthase (NRPS) were differentially expressed in ΔBcfrp1 compared with TB-31 (*p* < 0.05). Two genes that encoded Bcpks8 and Bcpks13 were downregulated at both the transcription and translation levels, and Bcpks13 was involved in the synthesis of dihydroxynaphthalene (DHN)-type melanin (Zhang et al., 2015). However, the Bcpks21 gene was upregulated at the translation level. The other two genes that encoded Bcpks11 and Bcpks20 were also upregulated at both the transcription and translation levels. However, their corresponding compounds are unknown. In addition, the gene for the non-ribosomal peptide synthase Bcnrps6 was upregulated at the transcription and translation levels.

The gene for sesquiterpene cyclase (Bcstc1, also designated Bcbot2) was decreased significantly at the transcription level in ΔBcfrp1 compared with that in TB-31 (*p* < 0.05). In addition, the other four genes (*bcbot1* and *bcbot3-5*) were significantly downregulated at both the transcription and translation levels in the botrydial (BOT) synthetic gene cluster. The results showed that the bocinic acid (BOA) synthetic genes of *bcboa3-5*, *bcboa10-12* and *bcboa15-17* were significantly downregulated at the transcription level. Moreover, the translation level of *bcboa2* also decreased, suggesting that *bcfrp1* could regulate the level of expression of the entire cluster.

### 3.9. DEGs and DEPs involved in development

We also analyzed the DEGs and DEPs involved in development based on the transcriptome and proteome data (Figure 7; Supplementary Table S6).

Leyronas et al. (2015) reported that *B.cinerea* is a heterothallic species, so there are two mating types (MAT1-1 and MAT1-2; Leyronas et al., 2015). In this study, we found that the transcription level of a characteristic MAT1-1 α-domain gene (Bcin01g02150), which also existed in the B05.10 (mating type MAT1-1), was downregulated in ΔBcfrp1 compared with that in TB-31 (*p* < 0.05). In addition, the translation levels of the probable gene for dipeptidyl-aminopeptidase B (Bcin15g01570) and G protein α-subunit Bcg1, which could be involved in the mating process and signaling, were downregulated in ΔBcfrp1. It was reported that three polyketide synthase genes including Bcpks13, hydroxynaphthalene reductase dehydrates (Bcbrn1) and scytalone dehydratase (Bcscd1) were involved in the synthesis of DHN melanin (Zhang et al., 2015). In this study, the *bcpks13* and *bcbrn1* genes were downregulated at both the transcription and translation levels in ΔBcfrp1 compared with those in TB-31 (*p* < 0.05), while the *bcscd1* gene was upregulated at both the transcription and translation levels.

### 3.10. DEGs and DEPs involved in plant cell wall degradation and the generation of reactive oxygen species

**Figure 8:**
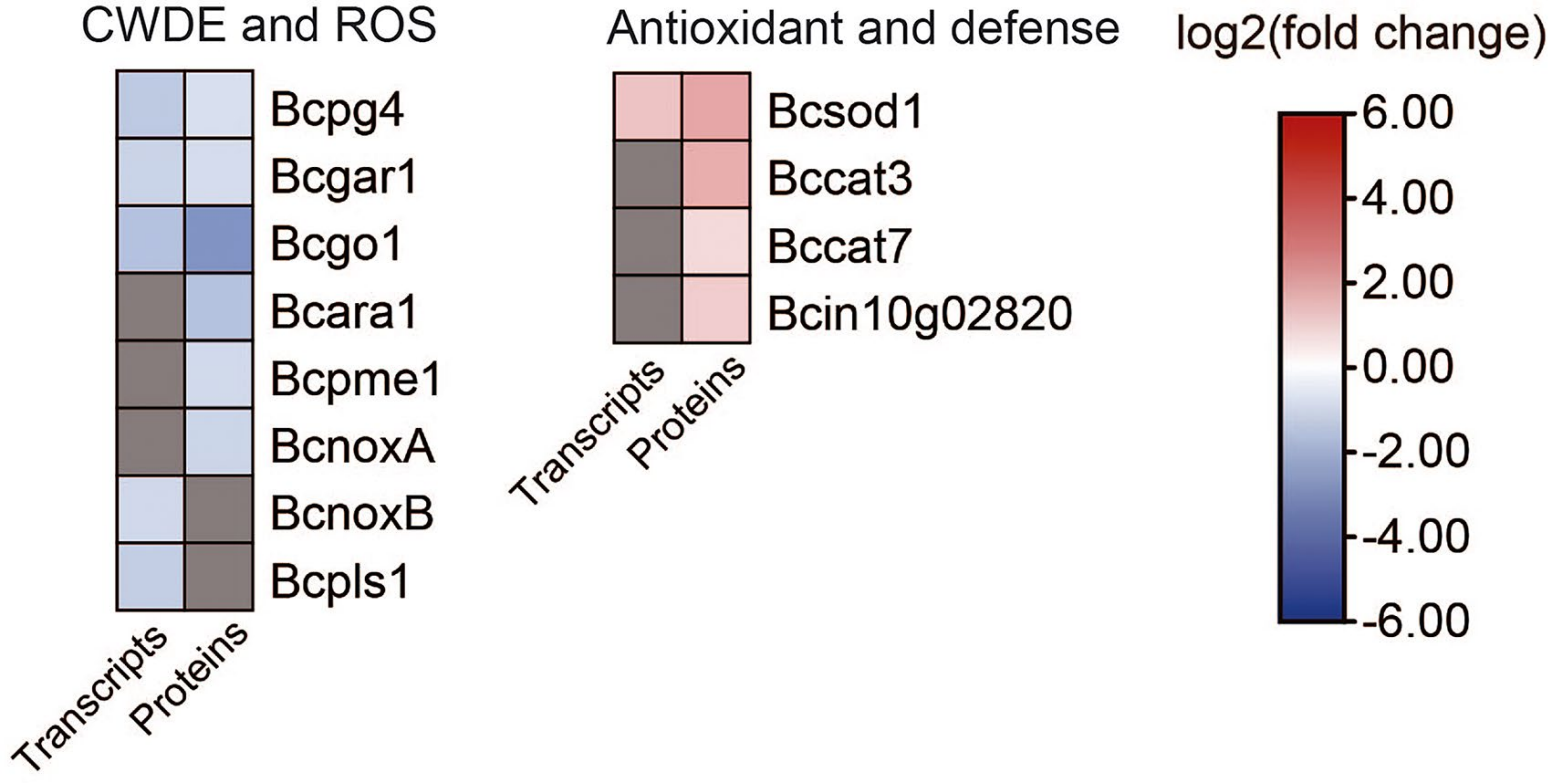
Heat map of DEGs and DEPs in plant cell wall degradation and reactive oxygen species generation between the *B. cinerea* TB-31 and ΔBcfrp1. The heat map was generated with the software TBtools (Toolbox for Biologists) V1.0971. The gray color means no significant difference at the transcription or translation level.

It was reported that the gene *fofrp1* is required for pathogenicity in *Fusarium oxysporum* (Jonkers et al., 2011). We analyzed the transcriptome and proteome data between *B. cinerea* TB-31 and ΔBcfrp1, and the results showed that; except for the genes involved in the synthesis of BOA and BOT toxins, there were also a considerable number of genes involved in pathogenicity that were differentially expressed (Figure 8; Supplementary Table S7).

While invading their hosts, many phytopathogenic fungi synthesize plant cell wall degrading enzymes (CWDE), for example xylanases, pectinases, and arabinanase (Amselem et al., 2011). Extracellular enzymes have been selected as potential virulence factors due to promoting the growth of hyphae. Three genes that encoded an endopolygalacturonase (Bcpg4), a galacturonate reductase (Bcgar1), and a glyoxal oxidase (Bcgo1) were downregulated at both the transcription and translation levels in ΔBcfrp1 compared with those in TB-31 (*p* < 0.05). Moreover, the two genes that encoded α-1,5-L-endo-arabinanase (Bcara1) and pectin methylesterase (Bcpme1), were downregulated at the translation level.

*Botrytis cinerea* is a necrotrophic pathogen that can generate reactive oxygen species (ROS) by enzymes. NADPH oxidases (Nox) are major enzyme systems that produce ROS, which can contribute to the virulence of the fungus. Siegmund et al. (2013) reported that the Nox including a tetraspanin (BcPls1) and two catalytic transmembrane subunits (BcNoxA, BcNoxB) were required for the virulence involved in the process of penetration in *B.cinerea* (Siegmund et al., 2013). In this study, the translation levels of *bcnoxA* were downregulated, while transcription levels of *bcnoxB* and *bcpls1* were downregulated in ΔBcfrp1 compared with TB-31 (*p* < 0.05), which could affect the penetration of ΔBcfrp1. In addition, the antioxidant enzymes can protect fungi by degrading the ROS produced by plants. In this study, the superoxide dismutase gene (Bcsod1) was upregulated at both the transcription and translation levels, and the catalase (Bccat3 and Bccat7) and glutathione S-transferase kappa 1 (Bcin10g02820) genes were upregulated at the translation level in ΔBcfrp1 compared with that of TB-31 (*p* < 0.05). Thus, we speculated that the upregulation of antioxidant enzymes could protect the fungal cell from the ROS.

### 3.11. Analysis of transcription factors and other genes associated with signal transduction

**Figure 9:**
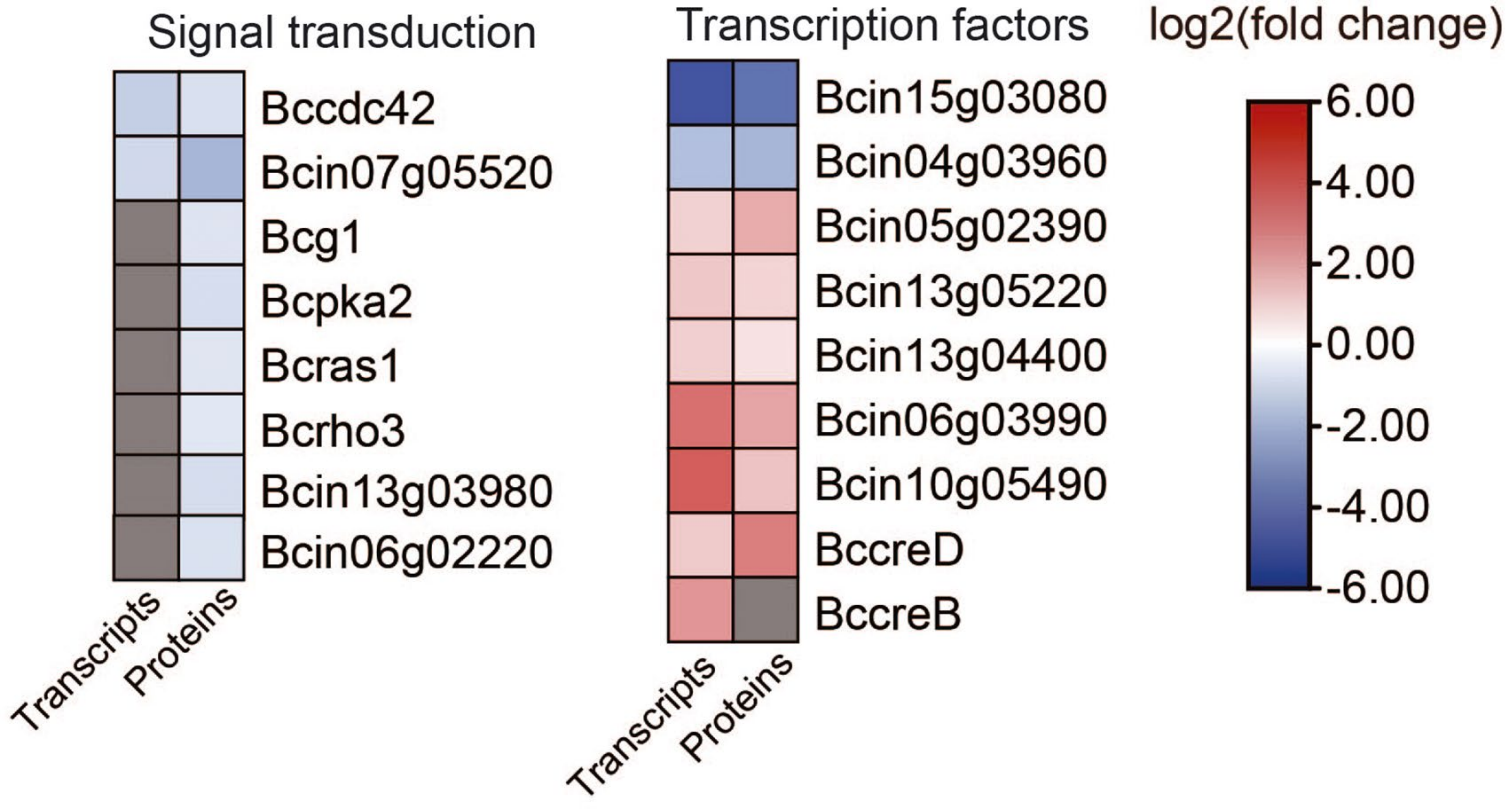
Heat map of DEGs and DEPs in transcription factors and signal transduction between the *B. cinerea* TB-31 and ΔBcfrp1. The heat map was generated with the software TBtools (Toolbox for Biologists) V1.0971. The gray color means no significant difference at the transcription or translation level.

Eight genes that are involved in signal transduction were differentially expressed in ΔBcfrp1 compared with TB-31 (*p* < 0.05; Figure 9; Supplementary Table S8). Two genes that encode the cell division control protein 42 (Bccdc42) and calmodulin (Bc4) were downregulated at both the transcriptional and translational levels. In addition, six genes were downregulated at the translational level, including the genes that encode heterotrimeric G α-subunit (Bcg1), PKA catalytic subunit (Bcpka2), medium Ras-like GTPase (Bcras1), small GTPase (Bcrho3), MAPK p21-activated kinase (Bcste20), and serine/threonine protein phosphatase pzh1.

In addition, nine genes that encode transcription factors (TFs) were differentially expressed in ΔBcfrp1 compared with TB-31 (*p* < 0.05; Figure 9; Supplementary Table S8). Of them, two genes that encode cutinase A (BccutA) and the regulatory subunit of protein phosphatase 1 Bcglc8, respectively, were downregulated at both the transcriptional and translational levels. Six genes that encoded TFs (Bcsfp1, Bctma46, Bcin13g04400, Bcku70, Bcku80, and BccreD) were upregulated at both the transcriptional and translational levels. In addition, a gene that encoded BccreB, a TF that is involved in the process of deubiquitinating CreA, was upregulated at the transcriptional level.

## 4. Discussion

The ubiquitin-proteasome system (UPS) is the main pathway for the degradation of intracellular protein in eukaryotes, which is composed of ubiquitin, ubiquitin-activating enzyme E1, ubiquitin-conjugating enzyme E2s, ubiquitin ligase E3s, 26S proteasome, and deubiquitinates (DUBs; Willems et al., 2004; Wu et al., 2022). SCFs complexes constitute a new class of E3 ligases, in which F-box protein is responsible for regulating its downstream target proteins by specific recognition and ubiquitination of protein substrates (Cardozo and Pagano, 2004; Johnk et al., 2016). The fungal F-box protein was initially found in *Saccharomyces cerevisiae*, and has been shown to play an important role in regulating a variety of cellular functions, including cell cycle regulation, nutritional sensing and fungal morphogenesis in fungi (Jonkers and Rep, 2009a; Sharma et al., 2021; Wu et al., 2022). For example, MUS-10 of *Neurospora crassa* is involved in cell senescence (Kato et al., 2010), Fbx23 and Fbx47 of *Aspergillus nidulans* participate in carbon catabolite repression (CCR), and Fbx50 (GrrA) is required for production of mature ascospores (De Assis et al., 2018). The F-box proteins are also required for virulence, like Fbx15 of *Aspergillus fumiga* and Fbp1 of *Cryptococcus neoformans* (Liu et al., 2011; Johnk et al., 2016). Frp1 is an F-box protein, which was initially found in *Fusarium oxysporum,* and its homologous proteins seem to show different functions in different fungi. Jonkers et al. (2011) reported that the F-box protein that encoded the gene *fofrp1* is necessary for virulence and carbon catabolism in *Fusarium oxysporum*, and the *B. cinerea* Bcfrp1 complemented into the ΔFofrp1 mutant restored pathogenicity (Jonkers et al., 2011). However, there is no effect on pathogenicity and sexual reproduction was impaired in the ΔBcfrp1 mutant produced by *B. cinerea* B05.10 (Jonkers et al., 2011). In our study, we found that a novel function of *bcfrp1* is required for the biosynthesis of ABA in *B. cinerea* TB-31. In this research, the mutant E154 that produced lower amounts of ABA revealed that the T-DNA insertion site was 1806 bp upstream of the predicted start codon of Bcfrp1. In addition, the deletion of Bcfrp1 had a significant effect on the biosynthesis of ABA in *B. cinerea* TB-31, and its yield decreased by 97% compared with TB-31 at 12 days. ABA yield of both E154-C and ΔBcfrp1-C restored to more than 80% by the complementation of *bcfrp1* gene. Although it did not reach the level of the control strain TB-31, which may be owing to the promoter sequence of *bcfrp1*, it was obvious that BcLAE1 was essential for ABA synthesis. To date, no reports have been published on the impact of F-box proteins on secondary metabolic regulation in *B. cinerea*.

To understand the evolution of Bcfrp1, we analyzed the conserved domain of F-box. The exact location of the F-box is 169–209 aa, defined as a receptor for ubiquitination targets (Supplementary Figure S3). A phylogenetic tree was constructed using the amino acids of the full length (Supplementary Figure S4), and resulted in the conclusion that orthologs of Bcfrp1 are present in fungi from six classes (Leotiomycetes, Sordariomycetes, Dothideomycetes, Eurotiomycetes, Pezizomycetes, and Xylonomycetes) belonging to the Pezizomycotina, and what’s interesting is that these fungi are pathogenic fungi, such as *Sclerotinia sclerotiorum*, *Hyaloscypha bicolor*, *Venustampulla echinocandica*, *Neurospora crassa*, *Trichoderma reesei*, *Fusarium graminearum*, *Fusarium oxysporum f.* sp. *lycopersici*, *Trematosphaeria pertusa*, *Xylona heveae*, *Tuber melanosporum*. Bcfrp1 orthologs were relatively close to *Botrytis porri*, *Botrytis byssoidea*, *Botrytis sinoallii*, *Botrytis fragariae* and *Sclerotinia sclerotiorum*, which might share an ancestor. It is not clear whether these strains have the capability to produce ABA. These homologous proteins were not characterized in the present study but may be involved in functional gene regulation related to pathogenicity in these pathogenic fungi. The mechanism underlying the functions of Bcfrp1 and its homologues are still to be elucidated.

It is apparent that different *frp1* mutant have phenotypic variations which indicate that Frp1 targets different proteins in different fungi. However, Jonkers and Rep (2009a,b) speculated that the main function of Frp1 does not depend on ubiquitination of targets (Jonkers and Rep, 2009a). They found Frp1 of *F. oxysporum* can bind to Skp1 in yeast two-hybrid and pull-down assays, but mutations in the F-box domain of Frp1 that impair binding to Skp1 do not affect the phenotype. Therefore, in order to better understand the downstream genes affected by Frp1, a stringent comparative transcriptome and proteome analysis was performed to identify differentially expressed genes between TB-31 and ΔBcfrp1, which would be very useful for novel target genes discovery. We found about 627 upregulated DEPs and 446 downregulated DEPs between TB-31 and ΔBcfrp1, and 552 proteins were shared between both DEPs and DEGs, suggesting the changes in the transcriptomic and proteomic level is very notable. This study will help us find targets that may be regulated by *bcfrp1* and expand our knowledge of the molecular basis by which *bcfrp1* regulates ABA biosynthesis in *B. cinerea*.

We first compared data from the transcriptome and proteome to analyze the DEGs related to the ABA biosynthetic pathway. In ΔBcfrp1 mutant, several genes involved in the biosynthesis of pyruvate and acetyl-CoA were upregulated at the transcription and/or translation level, which definitely favor the synthesis of acetyl-CoA. However, the levels of transcription and/or translation of the genes involved in IPP synthesis (MVA pathway), and the *bcaba1*-*4* genes involved in ABA synthesis from FPP were severely downregulated. As a result, we hypothesized that this is an important reason for the decrease of ABA biosynthesis in ΔBcfrp1 mutant. As Wang et al. (2018) reported the TF BcabaR1 directly regulate the promoter of ABA gene cluster (Wang et al., 2018). In this study, we found that the levels of transcription and translation of *bcabaR1* in ΔBcfrp1 did not change significantly when compared with TB-31. However, the levels of transcription of the entire ABA gene cluster were downregulated, so we speculated that there are other undiscovered regulatory mechanisms participating in the biosynthesis of ABA.

In addition to ABA, we also closely examined the other enzymes in secondary metabolism. The levels of transcription and translation of five key genes for enzymes were altered, including Bcpks8, Bcpks11, Bcpks13, Bcpks20, and Bcnrps6. The corresponding compounds of Bcpks8, Bcpks11 and Bcpks20 have not been identified. The level of translation of the sesquiterpene cyclase Bcbot2 gene was also downregulated, which is required for the virulence factor botrydial (BOT) biosynthesis. The biosynthetic gene cluster of BOT was similar to ABA, the remaining four genes in the BOT synthesis gene cluster (*bcbot1–5*) were downregulated at the levels of transcription and translation, while had no effect on TF Bcbot6. However, the deletion of Bcfrp1 resulted in the downregulation of Bcbot1-5 genes but not BcBot6, implying that Bcfrp1-induced clustering regulation could have another mechanism to bypass the TF. In addition, the level of transcription of nine genes in the bocinic acid (BOA) gene cluster decreased. In a previous study, we found that the regulator BclaeA is essential for the biosynthesis of ABA, which could affect the levels of transcription of the ABA, BOT, and BOA clusters (Wei et al., 2022). In this study, the levels of transcription and translation of *bclaeA* in ΔBcfrp1 did not change noticeably compared with TB-31, indicating that there are different regulatory mechanisms for BclaeA to synthesize ABA.

The downregulation of the BOA and BOT gene clusters may affect the virulence of *B. cinerea*. Dalmais et al. (2011) knocked out two genes, Bcbot1 and Bcbot2, involved in BOT production in the wild-type strain T4, which resulted in reduced virulence. In addition, they simultaneously knocked out the key genes that encode the enzymes Bcboa6 or Bcboa9 of BOA in the strain B05.10 led to the reduction of virulence (Dalmais et al., 2011). In this study, the level of expression for Bcboa6 or Bcboa9 did not change in ΔBcfrp1 compared with TB-31. However, several genes that encoded plant CWDE and NADPH oxidase enzymes, were downregulated at the transcription and/or translation levels in ΔBcfrp1, suggesting that Bcfrp1 plays key roles in regulating the level of expression of these genes.

Simultaneously, we found that the radial growth was decreased in the Bcfrp1 mutants. The mycelia of the ΔBcfrp1 transformants appeared white when compared with that of TB-31 owing to decreased expression of the key synthetic genes of melanin. The dihydroxynaphthalene (DHN) melanin biosynthetic pathway in *B. cinerea* comprises of the polyketide synthase PKS12 and PKS13, the hydrolase YGH1, tetrahydroxynaphthalene (TNH) reductase BRN1 and BRN2, and the scytalone dehydratase SCD1(Cheung et al., 2020). In our study, the *bcpks13* and *bcbrn1* genes are downregulated while the *bcscd1* is up-regulated in ΔBcfrp1. It was reported that deletion of the *bcpks13* gene resulted in albino conidia in *B. cinerea* (Zhang et al., 2015), and the *bcbrn1* deletion mutants was deficient in melanin biosynthesis, indicating the disruption of melanogenesis. Meantime, deletion of the *bcscd1* gene mutant also exhibited strong reduction in melanogenesis with no effects on other cellular processes. It was reported that the ΔBcscd1 mutant accumulated scytalone in the culture filtrate rather than the mycelium, and excessive scytalone appears to be self-inhibitory to the fungus (Chen et al., 2021). Therefore, the biological significance of up-regulation of *bcscd1* in ΔBcfrp1 is worth discussing, which may cause the decrease of scytalone accumulation. Some scientists believe that fungal melanin could also be perceived as a molecular pattern of pathogens by their hosts. In ascomycete rice blast fungus *Magnaporthe grisea*, DHN-melanin is a necessary component of the functioning appressorium (Jacobson, 2000). In human-pathogenic fungi *Aspergillus fumigatus*, DHN melanin specifically interferes with functions of host phagocytes, thus ensuring the virulence of the pathogen. Heinekamp et al. (2013) reported that DHN melanin and its precursors, like T4HN, and 1,8-DHN are also able to scavenge host-derived ROS in *A. fumigatus* (Leavitt et al., 2013). In TB-31, the effects of Bcfrp1, Bcpks13, Bcbrn1 and Bcscd1 on pathogenicity remains unclear. Jonkers et al. (2011) observed no effect on pathogenicity when Bcfrp1 in *B. cinerea* B05.10 was targeted for disruption (Jonkers et al., 2011). The *pks13* mutants also did not exhibit any changes in development or pathogenicity, and the effects of BRN1 deletion would be similar to other melanin biosynthesis genes and is nonessential for fungal development and pathogenicity. Therefore, the effect of Bcfrp1 and the functional genes regulated by Bcfrp1 on pathogenicity of *B. cinerea* TB-31 merits further study. In fact, *B. cinerea* TB-31 with substantially increased ABA yields have undergone many rounds of mutagenesis from a wild-type strain TBC-6, which was originally isolated from wheat stem, and leaf (Gong et al., 2014; Ding et al., 2016). Through observation, the mycelial growth of TB-31 is obviously weaker than that of TBC-6, B05.10 and T4, which may affect its colonization on the plant surface (data not shown). Thus, how to design experiments to evaluate the toxicity of TB-31 and ΔBcfrp1 mutant is the next challenge to consider.

Jonkers et al. (2011) compared the growth phenotypes of the ΔFrp1 mutants of *F. graminearum*, *F. oxysporum*, and *B. cinerea* on different carbon sources using a Biolog FF assay (Jonkers et al., 2011). The *F. oxysporum* ΔFofrp1 mutant grew significantly less on non-sugar carbon sources, which was very close to the previous growth phenotype on solid plates (Jonkers et al., 2009). In addition, the cell wall degradative genes and isocitrate lyase1 *icl1* were downregulated in the ΔFofrp1 mutant (Jonkers and Rep, 2009b). On the other hand, the ΔFgfrp1 mutant could normally grow on agar plates that contained various non-sugar carbon sources and polysaccharides. The *B. cinerea* B05.10 ΔBcfrp1 mutants grew well on any carbon source. The ‘cross-species’ complementation experiment with the Bcfrp1 of *B. cinerea* to replace the Fofrp1 of *F. oxysporum*, which led to the growth damage on non-sugar carbon source in ΔFofrp1 changed to the growth damage of sugar carbon source in the complementary mutant ΔFofrp1+ Bcfrp1. Jonkers et al. (2011) postulate that this difference is owing to the promoter sequence of *frp1* (Jonkers et al., 2011). In this study, we compared the colony diameters of TB-31, ΔBcfrp1, and ΔBcfrp1-C on CDA media for 6 days with several carbon sources (Figure 2D). As is shown in Figures 2D,E, after cultivation on CDA medium containing D-glucose and maltose for 6 days, the colony diameter of ΔBcfrp1 was significantly reduced to 25, and 37% of that of TB-31, respectively. Moreover, ΔBcfrp1 could not grow on CDA plates that contained sucrose. It is obvious that different *frp1* mutants have different phenotypes upon utilising different carbon sources, which is worthy of further study. We have also considered whether there is such a possibility that *bcfrp1* knockout affected the basic metabolism including carbon metabolism, and then indirectly affected the biosynthesis of ABA in TB-31. According to our observation, the different levels of expression of genes involved in the MVA pathway and the ABA biosynthetic gene cluster may lead to ABA deficiency in the ΔBcfrp1 strain. But we also found some genes involved in putative sugar transport and degradation, and the gene encoded hexokinase (Bcglk), were upregulated. Thus we do not know whether some products of sugar metabolism inhibit the pathway of ABA synthesis. Therefore, it is necessary to expand the screening range of carbon sources, detect ABA yields and the expression patterns of related genes under different carbon sources, and jointly analyses to gain insights into the carbon metabolism and the regulation of ABA synthesis in TB-31 and ΔBcfrp1.

In this research, we found that eight genes were downregulated in the signal transduction pathways, including *bcg1*, *bcras1*, *bcrho3*, *bccdc42*, *bcpka2*, *pzh1*, *bcste20*, and *bc4*. The function of the two genes that encoded serine/threonine-protein kinase Bcste20 and Calmodulin Bc4 remains unknown. As reported, the inactivation of *bcg1* (heterotrimeric Gpa2 homolog) caused a decrease in colonies on poor medium with sucrose as carbon source (Gronover et al., 2001), and inactivation by *bcras1* resulted in stunted hyphal growth (Minz Dub et al., 2013). Furthermore, when the *bcras1* gene from another small GTPase family was inactivated, the fungus grew less effectively (An et al., 2015), and the inactivation of *bccdc42* also caused a decrease in hyphal growth (Kokkelink et al., 2011; An et al., 2015). In this study, the *bcg1*, *bcras1*, *bcrho3*, and *bccdc42* genes were downregulated in ΔBcfrp1, which suggests that the decrease in hyphal growth could be related to the decrease in level of expression of these four genes. Fekete et al. (2004) reported that the metabolism of D-galactose except for *via* the Leloir pathway exists as an alternative pathway for hydrolytic D-galactose metabolism with L-sorbose as an intermediate in *A. nidulans* (Fekete et al., 2004). In this study, the glucose/galactose transporter gluP resulted in the downregulation of β-galactosidase, which catalyzes the conversion of lactose to galactose, and α-galactosidase, which catalyzes the conversion of D-galactose to sorbose, suggesting that the colony diameter of ΔBcfrp1 grown on lactose was similar to that of TB-31 and could be related to the alternative pathway of D-galactose metabolism. Ariño et al. (2019) reported that glycogen metabolism and cell cycle control in eukaryotes was regulated by the protein phosphatase PP1 (Ariño et al., 2019). In this study, the genes that encoded protein phosphatase pzh1 (glc7 homolog) and the regulatory subunit Bcglc8 were downregulated, suggesting that downregulation of the genes of glycogen metabolism could be related to the decreased expression of *pzh1* and *bcglc8*. In addition, carbon catabolite repression (CCR) is a global regulatory mechanism that is ubiquitous in microorganisms. In filamentous fungi, CCR is involved in catabolite responsive elements (*CreA*, *CreB*, *CreC*, and *CreD*) as well as the genes of cAMP and Protein Kinase (PKA) signal pathway (Adnan et al., 2017). The mutation of *cre1* in the *fofrp1* deletion mutant restored the impairments in the ΔFofrp1 mutant (Jonkers and Rep, 2009b), which indicates that the Fofrp1 and Cre1 proteins are both involved in the regulation of the CCR gene in *F. oxysporum*. In this study, we discovered that the regulation of *bccreA* and *bcsnf1* did not change significantly, whereas the expression of *bccreB and bccreD* increased in the ΔBcfrp1 generated from *B. cinerea* TB-31. Adnan et al. (2017) hypothesised that CreD was involved in the ubiquitination of CreA and CreB was involved in the deubiquitination of CreA, respectively (Adnan et al., 2017). The effect of *bccreB and bccreD* on the interaction of *bccreA* in *B. cinerea* requires further investigation.

In this study, 30 DEPs with unknown functions were revealed in the signal transduction pathways. We found about 2,661 upregulated DEGs and 1,647 downregulated DEGs, we also found about 627 upregulated DEPs and 446 downregulated DEPs between TB-31 and ΔBcfrp1, suggesting that the changes in the transcription and translation level is very notable. We hypothesise that the effect of Bcfrp1 on the change of expression level of a large number of genes may be amplified by regulating some signal transduction pathway.

In conclusion, we found that a novel function of *bcfrp1* is required for the biosynthesis of ABA in *Botrytis cinerea* TB-31. The deletion of *bcfrp1* resulted in the downregulation of the level of expression of the ABA biosynthetic gene cluster and the genes that participate in the mevalonic acid pathway. This study discovered the same regulatory pattern of Bcfrp1 targeting botrydial (BOT) and bocinic acid (BOA) gene clusters. In addition, DEGs and DEPs participating in the growth and carbon catabolite repression (CCR), and signal transduction in *B. cinerea* were identified based on the transcriptome and proteome data. Our future research will study the DEPs that are regulated by Bcfrp1 in more detail and search for potential targets in *B. cinerea*.

## Data availability statement

The data presented in the study are deposited in the ScienceDB (https://www.scidb.cn/detail?dataSetId=bf230977945c4b4785ec355293b23d79) and accession number (doi: 10.57760/sciencedb.06985).

## Author contributions

DC contributed to designing and performing the experiments, data analysis, and writing the manuscript. ZW helped with the data analysis. DS and HT helped with revision of the manuscript. All authors contributed to the article and approved the submitted version.

## Funding

This research received financial support from the National Natural Science Foundation of China (32070059). We acknowledge funding from the Technology Innovation Project of Chengdu Science and Technology Bure (2021-YF05-01197-SN) and Natural Science Foundation of Sichuan (2022NSFSC1634).

## Conflict of interest

The authors declare that the research was conducted in the absence of any commercial or financial relationships that could be construed as a potential conflict of interest.

## Publisher’s note

All claims expressed in this article are solely those of the authors and do not necessarily represent those of their affiliated organizations, or those of the publisher, the editors and the reviewers. Any product that may be evaluated in this article, or claim that may be made by its manufacturer, is not guaranteed or endorsed by the publisher.
