# Supplementary material for: Combined transcriptome and proteome analysis of Bcfrp1 involved in regulating the biosynthesis of abscisic acid and growth in Botrytis cinerea TB-31
Source: Front Microbiol. 2023 Jan 26;13:1085000. doi: 10.3389/fmicb.2022.1085000 (PMC9909433; doi:10.3389/fmicb.2022.1085000)
Supplement: Supplementary file 2 [file Data_Sheet_1.pdf]

Supplementary Material

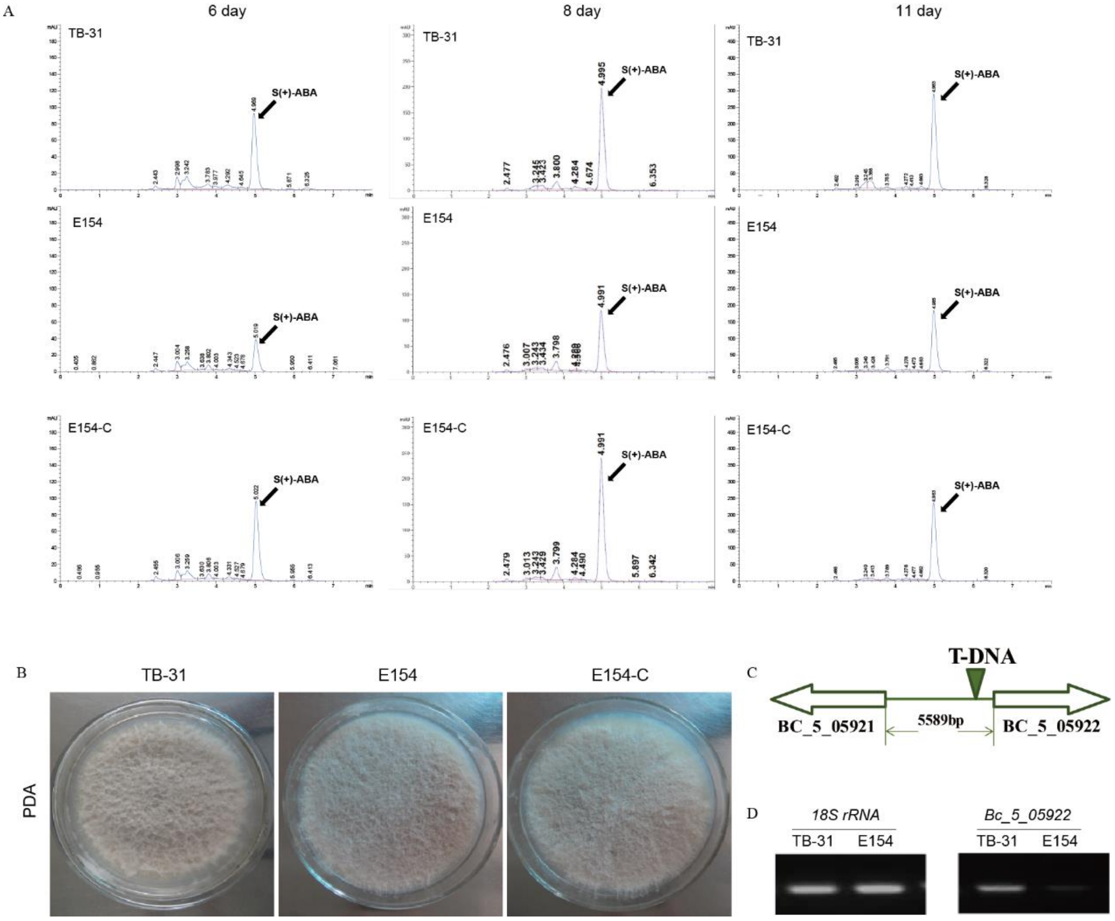

Figure S1. ABA synthesis-decreased mutant E154 was obtained through insertion mutagenesis. (**A**) ABA productivity of *B. cinerea* TB-31, mutant E154, mutant E154-C (E154 transformed with the cDNA of *bcfrp1*) grown on PDA for 6, 8 and 11 days, and a commercial S-(+)-ABA was used as the standard sample. (**B**) Phenotype of the parental strain *B. cinerea* TB-31, mutant E154 and E154-C (E154 transformed with the cDNA of *bcfrp1*) inoculated on PDA for 10 days. (**C**) Schematic representation of the T-DNA insertion site occurring upstream of BC_5_05922.（**D**）RT-PCR examining the transcription level of *bcfrp1* in mutant E154.

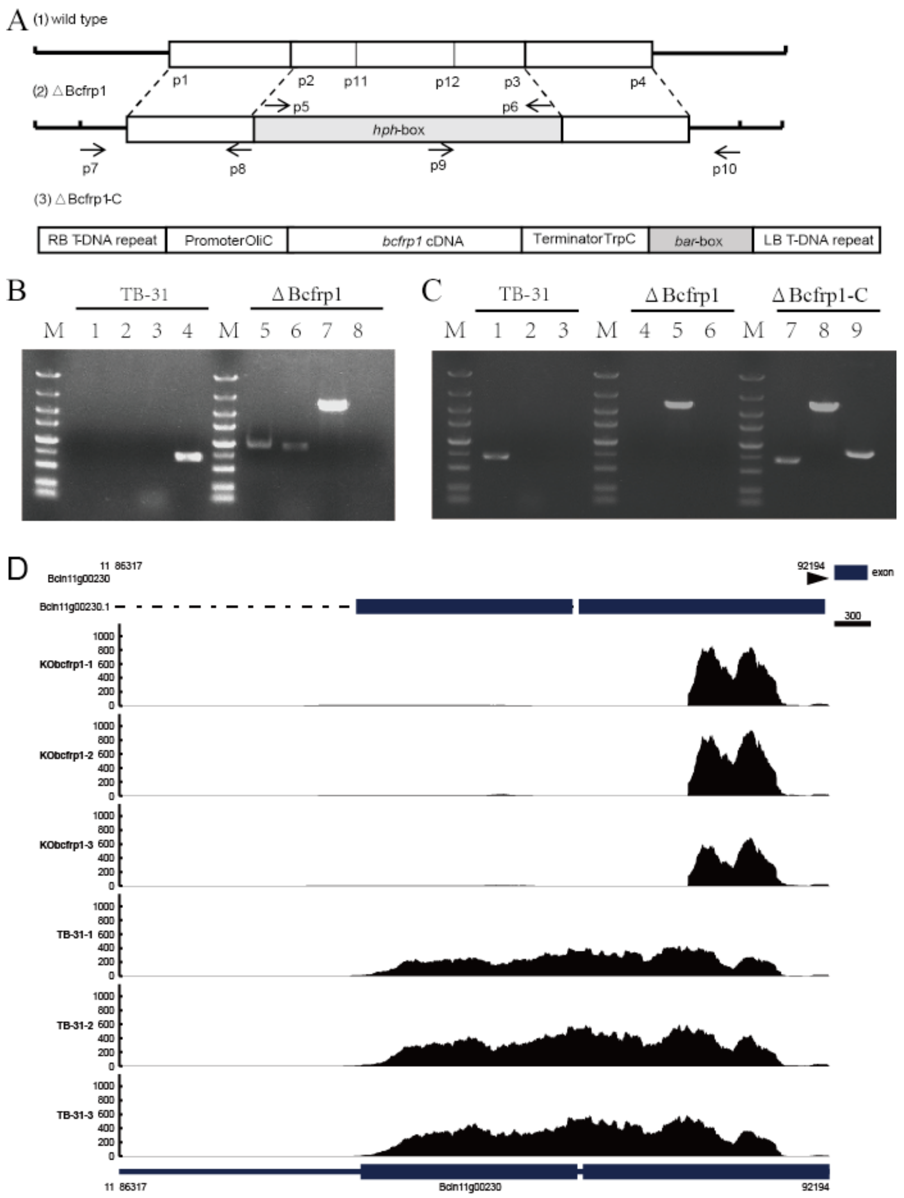

Figure S2. Identification of the ΔBcfrp1 and ΔBcfrp1-C mutant.

(A) Schematic representation of the *bcfrp1* deletion and complementation strategy. Hygromycin phosphotransferase fragment (*hph*) and glufosinate-ammonium resistance box are denoted by large light grey and grey. Arrows indicate the gene-specific primers. (B) PCR products for the *bcfrp1* gene replacement cassette. M:2000bp DNA Marker, Lane1/5: p7/p8 amplified the upstream of *bcfrp1*, Lane2/6: p9/p10 amplified the downstream of *bcfrp1*, Lane3/7: p5/p6 amplified Hph, Lane4/8: p11/p12 amplified the inside of *bcfrp1* ORF. (C) Based on gDNA PCR assay, M:5000bp DNA Marker, Lane1/4/7: p11/p12 amplified the inside of *bcfrp1* ORF, Lane2/5/8: p5/p6 amplified Hph, Lane3/6/9: *bar-in*-F/R amplified *bar*. (D) IGV result of *bcfrp1* between TB-31 and ΔBcfrp1.

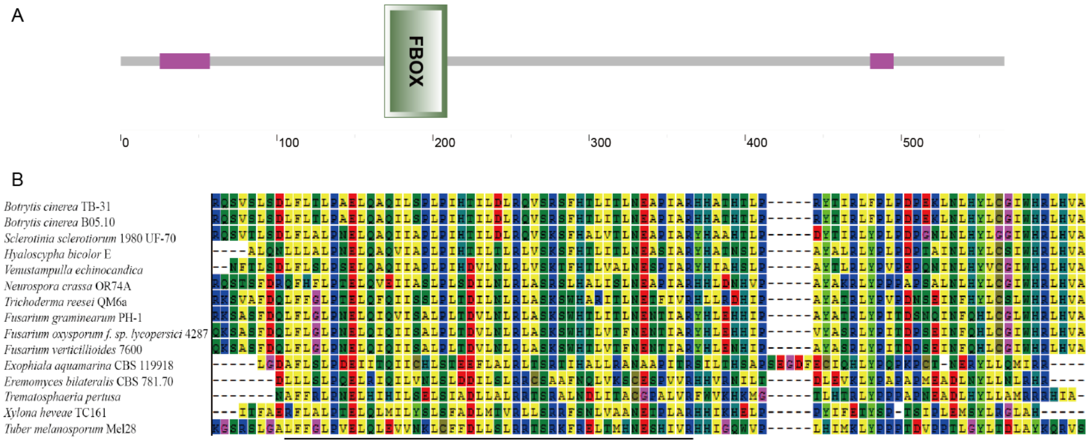

Figure S3. Analysis of conserved domain of F-box.

(A) Schematic representation of the Bcfrp1 domain analyzed by SMART. The exact location of the F-box is 169-209 aa, defined as a receptor for ubiquitination targets. The pink regions indicate that two low complexity regions, detected by the SEG program, were at position 25-57 and 500-515, respectively. (B) Alignment of Frp1 protein domains used for phylogenetic tree construction. An alignment of the various Frp1 proteins was created using the MEGA program, version 7.0.26. The alignment is based on the domain of the Frp1 proteins, which includes and extends beyond the F-box domain (underlined).

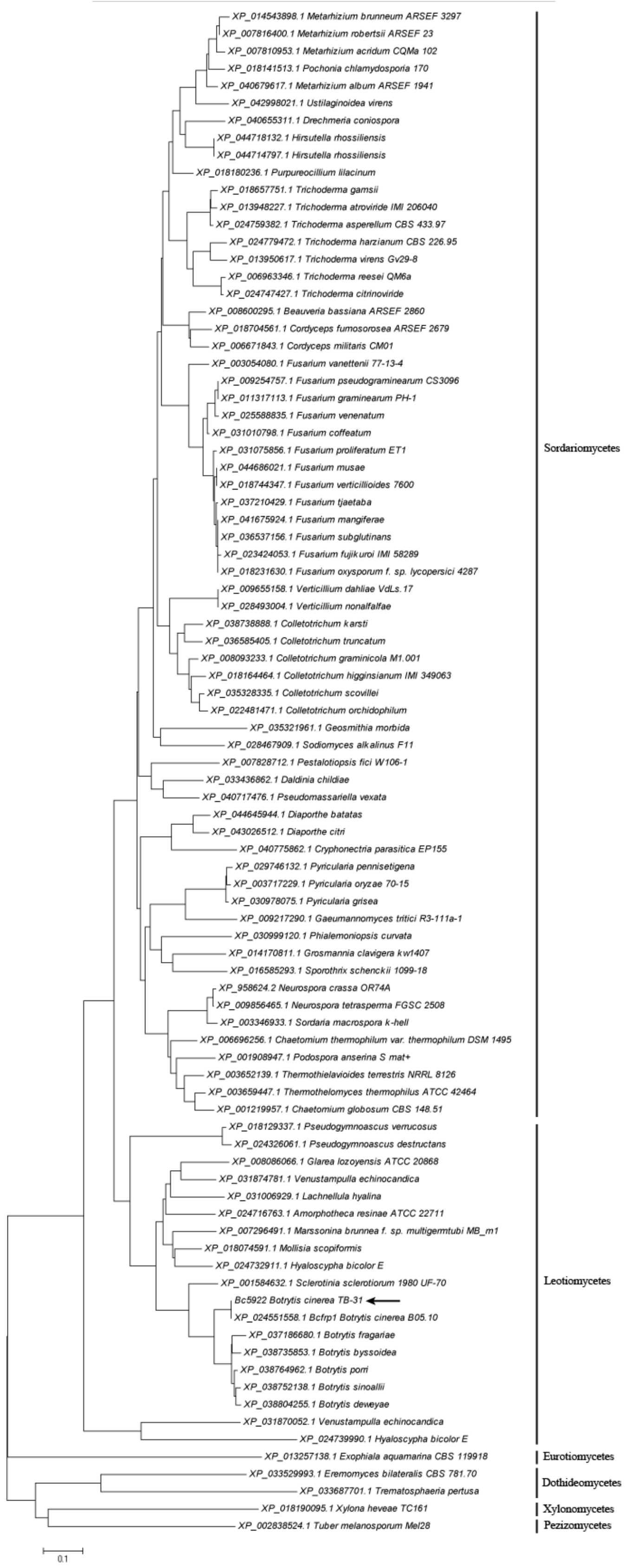

Figure S4. Phylogenetic analysis of F-box homologues in Ascomycetes.
The alignment was carried out with ClustalW and the MEGA program, version 7.0.26, was used for phylogenetic analysis using the maximum-likehood tree method.

Table S2. The result of TAIL-PCR.
The yellow indicates the T-DNA insertion site location at 1,806 bp upstream of the predicted start codon of BC_5_05922.

>GGACAGAGAAGAGGAGAGGaGaGGAGAGGAGAGGAGCGATGTTGTTTAC
GAAGCACATACCCATCTCTAAGAAGGACTAGTTGTTGACATGTCGTCTTAC
ATCGATGTATCATTTGGAGCCATATCGTGATTCTGATCTACTTGATGCCATCC
ATATCTTACCGTAAACATTCTCCTATGATCTTCAACCACGATTCAATCACGAG
CTCACAACTTCTAAGCGCAACAGACAGGGCTAGTTCTTGTATCTTCGATTC
AAGTATTCTACTTTGCCTGGGTCGCAGGTACATGTCCCAATATCAAAAGTTC
TTCCCCTCATCATCATCATCACCATCATCATCTCCCCACTCTCAATCCCTCTT
CATCGTCACACCCGAATCCCCATCCCCATCGCACCAATTCCATCAGTCATGT
TTtCCTCATTTCTCGTCAATTCATCAGTCATATTACCCAGCAGTCCAGGACCT
ACATTTCTCGTCTTACTTTATCCGGATCTCAATGTGCCTGGGTCCTGGATGTT
CCACAATTCCAATCAgTGGAGTGTGGAGTCTCCATAAAGACGCCGACCGAA
TGTTCCTTTGGTGCTAAACTCAAAGTGGGTATAGATTTGGATTGGGATCGGG
ATTGGGAAAATAAGATTTTGGAGAGATGTCCGTTGGGCGAATGGGGGGgga
GGATTGAATAGTTGGGCCGATAGAGAAGAAAATGAGATGTGTCTGTAGATG
GGATGAGGATGAGGATGAGGATGAGGATGACGAGgAGAGGATGAGAGGAG
AGAGACAAGAAGAATAGAGAAGAGGAACAaGCACTACTtaCTGTAGACAAG
CATATCAAGaGAAaGGAAagAAAGaGAGAATTCAACTTGTAGAGGAGAGAG
AGAGAgAGCGAGAGAGAGAGAGACTTTtACTTtAATCTAGAACTCAATCAG
CCATTGAATCTtCACTGAATCTTtACCGAAtCTTCGATCAATCTTCATtCAAAC
TTCACTGCAGTGAaCAAACCATTCCTTGATACTCGCGGTTAAaTATATATATAT
ATATATATATATATCATACATATAATaTAAGATATCCAGAACTTCCTCcTCCCCC
TCCCCcTTCTCCTCCTCCTcTTCcTCCCCcTACCCCCcTACCCCCTACCCCCTA
CcTCCCTCCCTACCGTGCTCACGCAGCTCGGCTTCATTATTGTGGTTAGGTA
GCATTTTTCAATCTACCATATCGGTATTACTATTAGCTGCTTTTACTAAGTTAC
TCCATACATAACAAGTGCGACTACCCAAACACTACTTGTAGTCTTGCAGTCT
GGTACTGGAAGAGGTTAAGATATTCAGAAAAATCATAAAACGTCAAGGGG
AAAACGTGTCTATCCCTTGAGATCCCACAATCCCATTGTCCCATTATCCCCA
TCTATTTCCTTCGTTAGTTCCCCCGATATACCGAATTCCTTTCTAGTCCCTTTA
TTTCTGGGAAGAGACGGGAGCGAGACGAGAAACGTATCTCCTCTGTCTATC
CCATCCGTCTATCCCATCAACCCAATCCATCTCAGCCGCCACACTTTCCACA
CTTTCCACCCTTTCCACCCTTACTCTTTTGAAGAGACAGAGAGAGCGAGAG
TAGAGAGAAAGGTTCCAGATCTTCCATCGTTCGTCAGTCGGTCGTTGTTTA
ACCTTTTCCTTTCTCCCTGCTCCCACTTCCACTTCCACATCATCGTCTTTTGT
CGATTTGTGTCTCTGGATCGATCTACAAACGGAAAGTCCGACGACACATGG
CTACTTGAATCCAACACTCCGACAACGACAACACAGCGACAACGGAAAAA
TCTAGACACTGCACACCACACGAAGAGAGAACTAGGATTGATGGAGGAAT
TGTCTTACCCATTTAACATCTTACGACATTAAGCCAGCAGCATAAATTCATCT
TCTTAAGTTTGTGGTGGTGTTATTCCTGTCTCTTTCAAAGTCACACTCGAAA
AAGGTCATCTCAGCAACAATAATCCGAACAACTCCAATTCCAGTCGATCGA
AACCATCACACTTTCGTTTTTCTTGGGCCCCCAAACAGAAGAAAGAAGAA
AATCTCTTGTATCTTCGTCTGGACTTTCTTTGGACTTTCTTTGGACATATCGA

TTATACCATCATCAACACAGGCAGACAGGATACCAAGGTACGAATACATACT
AAGACTTGTTATCTCGGTACTTTCTTCCATTTGACTCCGATCTCAACCCCAA
TCAATCCCTTATTGCTTGACCCTTCGACCCTTCAACCCTTCAACTTTCAACT
TTCGACTTCCGACTATCATACTATCCTCTCACACCTCTTTAACAGCCAACCC
ATCTTCCCAAACTTCTCTTGTCGATCTACATTGAGAACCTTTCCCTGCCTCT
CTCGAATAACCTGAATTGATATTGTTGACTTCTGAATCCTTTAACTGGTGTTT
TCAATAATGCATGAAGTTCAATCGTGCGACGTCGTTTCCTCAACTTTATTTC
AACTCGGCGCAATTTGGGCGGCCCTCCCTCTCGAAGAGCGTCGTCCATAAA
TCTTGATATGGTTTAACAATCACAAAGAACTTCAACTCTACGACCTATTGGT
ACTAAAGACCAAGCGCGGGGGAAGA

Table S3. DEGs and DEPs involved in putative sugar transports and degradation between *B.cinerea* TB-31 and ΔBcfrp1.

Gene ID	log_2FC of DEGs	log_2FC of DEPs	Gene annotation
**sugar transport**			
Bcin09g00150		2.150	Bchex1, Similar to MFS sugar transporter
Bcin05g07520	1.838	3.621	Bchex3, Similar to MFS sugar transporter
Bcin14g05360	1.090	1.441	Bchex5, Similar to MFS sugar transporter
Bcin12g06350	2.724	2.592	Bchex6, Similar to MFS sugar transporter
Bcin12g02300	1.098	2.273	glucose transporter rco-3
Bcin12g01880	1.749	3.074	glucose/galactose transporter gluP
Bcin06g06590	2.746	1.584	BcFrt1, Fructose proton symporter
Bcin10g04830	-1.045	-1.011	similar to sucrose transporter sut1
Bcin12g03830	-2.397		MAL11, maltose transporter
Bcin02g00080	-1.267		MAL61, maltose transporter
**sugar-degrading enzymes**			
Bcin02g01420	4.996	1.757	α-amylase
Bcin04g06250	3.446	1.440	α-amylase
Bcin14g00650	2.272	2.536	α-glucosidase
Bcin12g03390	2.416	2.228	α-glucosidase
Bcin04g04190	1.387	1.277	BcGs1, glycoamylase
Bcin08g02110	2.720	1.608	glycoside hydrolase family 5
Bcin16g03950	2.162	2.080	glycoside hydrolase
Bcin03g08710	3.370	3.407	glycoside hydrolase
Bcin14g00480	1.086	0.927	glycoside hydrolase
Bcin10g05710		0.605	glycosyl hydrolase family 32
Bcin03g02710		0.837	α-galactosidase
Bcin06g04500		0.978	β-galactosidase
Bcin09g04410	4.499	3.734	galactose oxidase
Bcin01g10310		-0.666	Bcgdb1, glycogen debranching enzyme
Bcin15g03620		-0.901	Bcgph1, glycogen phosphorylase
Bcin08g01920		-1.113	ADP-sugar bisphosphatase

Table S4. DEGs and DEPs involved in central metabolism and ABA biosynthesis between *B.cinerea* TB-31 and ΔBcfrp1.

Gene ID	log_2FC of DEGs	log_2FC of DEPs	Gene annotation
**glycolysis**			
Bcin09g04170	1.573	0.604	Bcglk, hexokinase
Bcin09g03850	1.108	0.852	Transaldolase
Bcin01g10330	1.487	0.785	DAHP synthetase I
Bcin07g03760	-1.467		fructose-bisphosphate aldolase
Bcin08g05220		-0.862	Triosephosphate isomerase
Bcin15g02120		-0.608	Glyceraldehyde-3-phosphate dehydrogenase
**PDH-bypass**			
Bcin02g06580	3.896	2.206	pyruvate decarboxylase
Bcin13g05810		1.489	aldehyde dehydrogenase (NAD+)
**PDH**			
Bcin02g06400		-0.594	dihydrolipoamide dehydrogenase
**TCA**			
Bcin09g00650	1.576	1.771	Bccit3, citrate synthase
Bcin13g02430	-1.570	-0.974	ATP citrate lyase
Bcin02g03080	-1.754	-0.973	Succinate dehydrogenase
Bcin16g00630	-1.504	-0.913	Bcpck1, phosphoenolpyruvate carboxykinase
**GYC**			
Bcin09g00660		0.688	Bcicl1, Isocitratelyase1
Bcin16g03120	1.234	0.606	Malate dehydrogenase
**fatty acids β-oxidation**			
Bcin03g05840		-0.778	enoyl-CoA hydratase
Bcin07g04170		-0.866	AMP-dependent synthetase/ligase
**Terpenoid Skeleton Biosynthesis**			
Bcin05g07430	-2.693	-0.844	Bcerg10, Acetyl-CoA C-acetyltransferase
Bcin11g00330	-1.021	-1.109	Bcerg13, HMG-CoA synthase
Bcin05g03770		-0.737	Bcerg12, Mevalonate kinase
Bcin04g01890	-1.045		Bcmvd1, MVA kinase
**ABA biosynthesis**			
Bcin08g03850		-0.906	Bcaba1, Cytochrome P450 monooxygenase, in abscisic acid gene cluster
Bcin08g03840		-1.200	Bcaba2, Cytochrome P450 monooxygenase, in abscisic acid gene cluster
Bcin08g03880		-1.487	Bcaba3, In abscisic acid gene cluster

Table S5. DEGs and DEPs involved in secondary metabolism between *B.cinerea* TB-31 and ΔBcfrp1.

Gene ID	log_2FC of DEGs	log_2FC of DEPs	Gene annotation
**PKS**			
Bcin07g02920	-1.937	-3.049	Bcpks8, Polyketide synthase
Bcin03g08050	-2.296	-1.928	Bcpks13, Polyketide synthase
Bcin14g01290	4.716	3.701	Bcpks11, Polyketide synthase
Bcin04g00640	1.400	2.051	Bcpks20, Polyketide synthase
Bcin05g08400		0.624	Bcpks21, Polyketide synthase
**NRPS**			
Bcin01g03730	2.214	2.122	Bcnrps6, Nonribosomal peptide synthase
**Botrydial (BOT)**			
Bcin12g06380	-2.764	-3.593	Bcbot1, Benzoate 4-monooxygenase cytochrome p450
Bcin12g06390	-2.712		Bcbot2, Bcstc1, Sesquiterpen cyclase
Bcin12g06400	-2.906	-2.968	Bcbot3, Cytochrome P450 monooxygenase
Bcin12g06370	-2.619	-2.286	Bcbot4, Cytochrome P450 monooxygenase
Bcin12g06410	-3.486	-1.950	Bcbot5, Acetyl transferase
**Botcinic acid (BOA)**			
Bcin01g00020		-0.834	Bcboa2, flavin-binding monooxygenase-like
Bcin01g00030	-1.856		Bcboa3, cytochrome P450
Bcin01g00040	-1.418		Bcboa4, cytochrome P450
Bcin01g00050	-1.257		Bcboa5, alcohol dehydrogenase GroES-like domain
Bcin01g00100	-2.507		Bcboa10, thioesterase domain
Bcin01g00110	-1.081		Bcboa11, transferase family
Bcin01g00120	-1.350		Bcboa12
Bcin01g00140	-1.290		Bcboa15, fungal specific transcription factor domain
Bcin01g00150	-2.190		Bcboa16, NAD dependent epimerase/dehydratase family
Bcin01g00160	-1.270		Bcboa17, short chain dehydrogenase

Table S6. DEGs and DEPs involved in development between *B.cinerea* TB-31 and ΔBcfrp1.

Gene ID	log_2FC of DEGs	log_2FC of DEPs	Gene annotation
Bcin01g02150	-1.106		Mating-type protein MAT1-1
Bcin15g01570		-0.672	probable dipeptidyl-aminopeptidase B
Bcin05g06770		-0.725	Bcg1, Heterotrimeric Galpha subunit
Bcin03g08050	-2.296	-1.928	Bcpks13, Polyketide synthase
Bcin04g04800	-3.915	-1.608	Bcbrn1, Tetrahydroxynaphtalene (THN) reductase

Table S7. DEGs and DEPs involved in cell wall degrading enzymes and reactive oxygen species between *B.cinerea* TB-31 and ΔBcfrp1.

Gene ID	log_2FC of DEGs	log_2FC of DEPs	Gene annotation
Bcin03g01680	-1.428	-0.836	Bcpg4, Endopolygalacturonase
Bcin06g04660	-1.148	-0.907	Bcgar1, Galacturonate reductase
Bcin06g01930	-1.612	-2.904	Bcgo1, Glyoxal oxidase
Bcin02g07700		-1.608	Bcara1, Alpha-1,5-L-endo-arabinanase
Bcin08g02970		-0.983	Bcpme1, Pectin methyl esterase
Bcin05g00350		-1.108	BcnoxA, NADPH oxidase
Bcin02g04930	-1.026		BcnoxB, NADPH oxidase
Bcin09g06130	-1.293		Bcpls1, Tetraspanin
Bcin03g03390	1.320	1.965	Bcsod1, Superoxide dismutase
Bcin06g04520		1.807	Bccat3, Catalase
Bcin09g04400		0.850	Bccat7, Catalase
Bcin10g02820		1.127	glutathione S-transferase kappa 1

Table S8. DEGs and DEPs involved in transcription factors and genes associated with signal transduction between *B.cinerea* TB-31 and ΔBcfrp1.

Gene ID	log_2FC of DEGs	log_2FC of DEPs	Gene annotation
**Signal transduction**			
Bcin13g00090	-1.278	-0.799	Bccdc42, Cell division control protein 42
Bcin07g05520	-1.006	-1.945	Bc4, Calmodulin
Bcin05g06770		-0.725	Bcg1, Heterotrimeric Galpha subunit
Bcin08g03910		-0.863	Bcpka2, PKA catalytic subunit
Bcin12g05760		-0.665	Bcras1, Ras-like GTPase
Bcin06g04390		-0.615	Bcrho3, Small GTPase
Bcin13g03980		-0.886	Bcste20, MAPK: PAK
Bcin06g02220		-0.778	protein phosphatase pzh1, (Glc7 homolog of *Saccharomyces cerevisiae*)
**Transcription factors**			
Bcin15g03080	-4.900	-3.890	BccutA, Cutinase A
Bcin04g03960	-1.679	-1.949	Bcglc8, Regulatory subunit of protein phosphatase 1
Bcin05g02390	1.044	1.839	Bcsfp1, Regulates transcription of ribosomal protein and biogenesis genes
Bcin13g05220	1.228	0.967	Bctma46, Protein of unknown function that associates with translating ribosomes
Bcin13g04400	1.061	0.663	Helicase-like, DEXD box c2 type + ATP-dependent helicase, C-terminal
Bcin06g03990	3.217	1.999	Bcku70, Protein involved in DNA reparation
Bcin10g05490	3.700	1.346	Bcku80, Protein involved in DNA reparation
Bcin13g02620	1.172	2.865	BccreD, Arrestin C-terminal-like domain
Bcin07g04660	2.323		BccreB, Ubiquitin-specific peptidase
